# Teacher Coaching and Consultation with Active Components for the Implementation of Social–Emotional Learning Programs in Basic Education: A Systematic Review with Thematic Synthesis

**DOI:** 10.3390/bs16091513

**Published:** 2026-08-28

**Authors:** Juan Diego Dávila Cisneros, Lina Iris Palacios-Serna, Alfredo Puican Carreño, Claudia Virginia Cortez-Chavez, Mary Roxana Salazar Calderón

**Affiliations:** 1 School of Sociology, College of Historical, Social, and Educational Sciences, Universidad Nacional Pedro Ruiz Gallo, Lambayeque 14013, Peru; 2 Graduate School, Faculty of Education, Universidad Privada Antenor Orrego, Trujillo 13008, Peru; lpalacioss1@upao.edu.pe; 3 School of Education, College of Historical, Social, and Educational Sciences, Universidad Nacional Pedro Ruiz Gallo, Lambayeque 14013, Peru; apuican@unprg.edu.pe; 4 School of Psychology, Faculty of Health, Universidad San Ignacio de Loyola, Lima 15024, Peru; ccortez@usil.edu.pe; 5 Psychology Program, Faculty of Health Sciences, Universidad San Ignacio de Loyola, Lima 15024, Peru; msalazarc@usil.edu.pe

**Keywords:** teacher coaching, instructional consultation, social–emotional learning, implementation fidelity, practice-based coaching, professional development, mixed-methods systematic review, thematic synthesis

## Abstract

Background: Teacher coaching is a central driver of implementation science, yet no recent review has centered the active components of coaching and consultation, rather than social-emotional learning (SEL) programs themselves, as its primary object of synthesis. (“Basic education” is used throughout this manuscript as the English rendering of the Latin American usage of the term, corresponding to ISCED 2011 levels 0–2: early childhood, primary, and lower-secondary education; it does not include upper-secondary or tertiary education.) This mixed-methods systematic review with thematic synthesis examined how coaching and consultation models with explicit active ingredients (modeling, guided practice, reflection, goal setting, and performance feedback) affect implementation fidelity, dosage, and quality, and students’ socioemotional, behavioral, and academic outcomes. Methods: Following PRISMA 2020 and a prospectively registered protocol (OSF M3846), we searched Scopus, Web of Science, and ERIC, supplemented by snowballing and grey literature searches. Of 341 screened records, 36 studies (2010–2026) met eligibility criteria and underwent data extraction and MMAT quality appraisal by independent reviewers (κ ≥ 0.84). A convergent segregated mixed-methods synthesis integrated qualitative thematic analysis with narrative quantitative synthesis. Results: Four interdependent macro-themes emerged: the systemic and individual conditions enabling coaching; its active technical-relational architecture; fidelity–adaptation tensions when scaling programs across cultures; and a translational cascade whereby high-fidelity coaching is associated with stronger proximal teacher practice and classroom climate, with academic gains emerging later and inconsistently. Conclusions: Coaching appears most effective when active technical components combine with a non-evaluative consultative alliance under supportive school conditions. We propose adaptive fidelity as a framework reconciling technical rigor with cultural responsiveness, relevant for scaling coaching to lower-resource, culturally diverse contexts, including the Global South.

## 1. Introduction

Social-emotional learning (SEL) constitutes one of the most influential contemporary frameworks for promoting students’ holistic development in school settings. Defined as the process through which children, adolescents, and adults acquire and apply the knowledge, skills, and attitudes needed to manage emotions, set goals, maintain positive relationships, and make responsible decisions, SEL has become a priority component of basic education in educational systems worldwide (i.e., ISCED 2011 levels 0–2; UNESCO, 2012) (Durlak et al., 2011; Cipriano et al., 2023). Durlak and colleagues’ seminal meta-analysis, based on 213 universal interventions involving more than 270,000 K–12 students, found gains on the order of 11 percentile points in academic performance among SEL program participants, together with significant improvements in social skills, attitudes, and behavior (Durlak et al., 2011). More than a decade later, Cipriano et al.’s (2023) meta-analysis, synthesizing 424 studies from 53 countries and 252 discrete SEL interventions, confirms and extends that evidence: participating students show substantive benefits in social-emotional competencies, school climate, peer relationships, and academic achievement.

This accumulation of evidence coexists, however, with a persistent finding: the magnitude of SEL effects depends on the quality with which programs are implemented in the classroom, which introduces substantial variability across schools, teachers, and contexts (Cipriano et al., 2023; Ulla & Poom-Valickis, 2023). Ulla and Poom-Valickis’s (2023) review synthesizes the contextual factors that facilitate SEL program implementation and concludes that program support (particularly modeling during coaching and the teacher–coach relationship) ranks among the most consistent predictors of implementation quality, outweighing many of the classic variables associated with teacher profile. This finding aligns with the broader perspective of implementation science, which positions coaching as a central engine for ensuring that evidence-based practices are delivered as designed (Snyder et al., 2015).

Teacher coaching, understood as individualized professional support that is practice-embedded and oriented toward improving instruction, has become established as an alternative to traditional teacher-training models based solely on one-off workshops. Kraft et al.’s (2018) causal meta-analysis, synthesizing 60 studies with experimental or quasi-experimental designs, reports pooled effect sizes for coaching of 0.49 standard deviations on instructional practice and 0.18 on student achievement, placing coaching among the professional development strategies with the strongest available evidence. Kraft and colleagues nonetheless warn of a systematic decline in these effects as programs scale up, anticipating a core tension in the field: what works under optimal efficacy conditions does not necessarily hold when translated into the routine implementation of entire school systems.

Within the specific domain of school-based SEL, this general evidence on coaching has translated into models with explicit conceptual and empirical foundations. Snyder et al.’s (2015) Practice-Based Coaching operationalizes coaching as an iterative cycle of collaborative goal planning, situated observation, guided reflection, and performance feedback, and is one of the most widely used frameworks in early-childhood SEL programs such as the Pyramid Model. In elementary education, Doyle et al. (2023) quasi-experimentally evaluated, across 91 classrooms in urban schools, the integration of the 4Rs curriculum (Reading, Writing, Respect and Resolution) with the MyTeachingPartner coaching model, reporting academic and behavioral improvements associated with structured coaching support relative to 4Rs implementation without it. At the systemic level, Domitrovich et al. (2025), through a cluster-randomized trial across 28 low-performing urban elementary schools, examined the effects of the CASEL School Guide, which adds monthly coaching to the PATHS curriculum, on organizational capacity to sustain SEL at the school level.

Despite this convergent evidence, the field presents unresolved tensions that justify a dedicated synthesis effort. First, evidence on student-level effects is less consistent than evidence on teacher implementation: coaching tends to improve the fidelity and quality of teaching, but its translation into sustained socioemotional or academic outcomes is heterogeneous and, in some cases, null (Doyle et al., 2023; Domitrovich et al., 2025). Second, primary studies operationalize what constitutes coaching in disparate ways: modeling and feedback, external school mental-health consultation, or brief or digital formats. This heterogeneity of active components precludes a clear accumulation of evidence without a dedicated systematization (Snyder et al., 2015; Ulla & Poom-Valickis, 2023). Third, existing reviews, including the major SEL meta-analyses (Durlak et al., 2011; Cipriano et al., 2023) and the review of contextual implementation factors (Ulla & Poom-Valickis, 2023), treat coaching as one factor among several, without centering it as the primary object of synthesis. Compounding this, the reviewed bodies of literature are dominated by English-speaking and European contexts, with marginal representation from the Global South, which limits the transferability of conclusions to educational systems such as those in Latin America.

In light of this landscape, the present review with thematic synthesis addresses a methodologically justified gap: no review published in the last five years has centered, as its primary object of synthesis, the active components of teacher coaching and consultation as strategies for supporting the implementation of SEL interventions in school settings. The review question was structured using the PICO framework as follows: Population, teachers in basic education implementing SEL programs or social-emotional teaching practices; Intervention, coaching or consultation models with explicit active ingredients (modeling, guided practice, reflection, goal setting, and performance feedback); comparison, less specific, lower-intensity, or pre-service-training-only supports; outcome, fidelity, dosage, and quality of implementation, as well as students’ socio-emotional, behavioral, and academic outcomes. In short: how do coaching or consultation models with explicit active ingredients affect implementation and student outcomes, relative to less specific or lower-intensity support? The overall objective is to systematically synthesize the available qualitative and mixed-methods evidence on these models, following the PRISMA 2020 guidelines and appraising methodological quality with the Mixed Methods Appraisal Tool (MMAT). Given the methodological and contextual heterogeneity expected in the corpus, no meta-analysis is anticipated. Ultimately, this synthesis seeks to clarify which coaching characteristics are most consistently associated with implementation fidelity and sustainability, and to derive transferable guidance for designing teacher support systems across diverse educational contexts, including those underrepresented in the international evidence base.

## 2. Materials and Methods

### 2.1. Protocol and Registration

This systematic review was conducted following a prospectively registered protocol, developed in accordance with the PRISMA-P 2015 statement (Moher et al., 2015) and registered on the Open Science Framework prior to the selection of identified records (OSF project M3846; https://doi.org/10.17605/OSF.IO/M3846). The protocol (version 1.7) documents seven rounds of amendments (v1.1–v1.7); the two most substantial amendments broadened the study-design eligibility criteria to include quantitative designs (Section 2.2) and specified a convergent segregated mixed-methods synthesis (Section 2.7). Both amendments were adopted during full-text selection, after the team determined that the originally stated design criterion, limited exclusively to qualitative or mixed methods, was systematically excluding several of the most methodologically rigorous studies fundamental to the field of teacher coaching for SEL implementation (e.g., cluster-randomized efficacy trials such as Domitrovich et al., 2025). This decision was made partway through, rather than after, full-text screening (Phase 2 was roughly one-third complete), and was motivated by the pattern of exclusions by study design (entire categories of quantitative studies being excluded regardless of their content or findings), not by knowledge of which specific studies’ results were favorable or unfavorable to coaching; reviewers were, of course, exposed to each record’s reported findings as an unavoidable part of full-text eligibility assessment, so we cannot claim the amendment decision was made under formal outcome-blinding. To guard against this risk, the eligibility criteria themselves were not altered for any individual study after that study had already been screened under the broadened criteria, and the decision to broaden eligibility was applied uniformly, retrospectively, to all records already screened and prospectively to those not yet screened, rather than being applied selectively. We treat the possibility of residual selection bias from this protocol change as a limitation (Section 4.4). The substantive amendments, including the two described above, are reproduced with their stated justification in Supplementary Table S12; the complete version-by-version amendment log (v1.1–v1.7), with dates and justifications for each revision, is available in the OSF project wiki. This review is reported in accordance with the PRISMA 2020 statement (Page et al., 2021).

### 2.2. Eligibility Criteria

Inclusion criteria were defined a priori using the PICO framework (Table 1).

Eligible study designs initially included qualitative designs (phenomenology, case study, ethnography, grounded theory) and mixed-methods designs (convergent, explanatory sequential, exploratory sequential, and embedded). Qualitative and mixed-methods designs were prioritized from the outset because the review question centers on how coaching and consultation operate, that is, the mechanisms, relational processes, and contextual conditions of implementation, rather than solely on whether they produce an effect of a given magnitude; qualitative evidence uniquely captures these mechanisms in a way that effect sizes alone cannot (Section 2.7.1). During full-text selection, the protocol (v1.7) was amended to additionally admit quantitative designs, namely randomized controlled trials, quasi-experimental designs (with or without a comparison group), single-case experimental designs, and descriptive/correlational designs, once it became clear that this criterion, rather than substantive relevance, was driving the exclusion of foundational studies in this literature. Eligible records were published between 1 January 2010 and 23 June 2026. No language restriction was applied; records in languages the team did not command were translated during full-text analysis using professional or AI-assisted translation tools, and translations were archived for audit purposes. Eligible publication types were peer-reviewed journal articles, publisher-reviewed book chapters, doctoral theses, and technical reports from research organizations subject to external review (e.g., MDRC, RAND, SRI International), provided the full text was available for assessment.

One deliberate exception to the population criterion was made: Longhitano (2021), a lesson-study coaching intervention with high-school (ISCED 3) mathematics teachers, was retained as a single extension case beyond the review’s core ISCED 0–2 population. It was kept because its coaching model met all six active-component criteria with unusual methodological detail, and because excluding the corpus’s only secondary-level case would have left the review silent on whether the active-ingredients framework extends beyond basic education as narrowly defined. This case is treated as a sensitivity/extension observation rather than as evidence for the secondary level per se; its effect on the synthesis is revisited in Section 4.4 (Limitations).

A seven-code exclusion catalog (E1–E7), summarized in Table 2, was applied systematically at both the title/abstract screening stage (Phase 1) and the full-text screening stage (Phase 2).

### 2.3. Information Sources and Search Strategy

Searches were conducted across three bibliographic databases: Scopus (Elsevier; TITLE-ABS-KEY field), Web of Science Core Collection (Clarivate; “Topic” field; SSCI and ESCI indexes), and ERIC (Institute of Education Sciences; native eric.ed.gov interface). The database search was supplemented with backward snowballing from the reference lists of included studies, a forward citation search in Google Scholar to identify key studies, and a grey literature search of doctoral thesis repositories (ProQuest Dissertations & Theses; OATD) and Latin American institutional repositories (Alicia; La Referencia) to mitigate geographic publication bias. Searches were conducted between 22 and 23 June 2026.

The canonical search string combined four conceptual blocks derived from the PICO model (terms related to SEL, terms related to teachers and training, terms related to coaching and consultation, and terms related to implementation and outcomes) with a context block (from early childhood through secondary education) and a “NOT” block excluding terms related to pre-service teacher training and higher education, adapted to the syntax of each database. The full canonical string, as executed in Scopus, was:

TITLE-ABS-KEY((“social-emotional learning” OR SEL OR “social emotional learning” OR “social-emotional competenc*” OR “social emotional competenc*” OR “social-emotional teaching” OR “emotion* coaching” OR “school-based SEL”) AND (teacher* OR educator* OR “school staff” OR classroom*) AND (coach* OR consultation OR consult* OR “practice-based coaching” OR “instructional coaching” OR “performance feedback” OR modeling OR modelling OR “guided reflection” OR “goal setting” OR observation OR “guided practice”) AND (“implementation fidelity” OR fidelity OR implementation OR dosage OR adherence OR quality OR “program implementation” OR “implementation quality” OR “student outcome*” OR “academic outcome*” OR “behavior* outcome*” OR “social-emotional outcome*”) AND (school* OR preschool* OR kindergarten OR “early childhood” OR elementary OR “middle school” OR “secondary school”) NOT (“preservice teacher*” OR “teacher candidate*” OR universit* OR college*))

Equivalent strings, adapted to native syntax, were executed in Web of Science (TS=) and ERIC. The full per-database strings, execution dates, and raw record counts, documented in accordance with PRISMA-S (Rethlefsen et al., 2021), are reproduced verbatim in Supplementary Table S11, together with technical notes on adapting the canonical string to each database’s native syntax; original export files (.ris/.nbib) are archived in the OSF repository.

### 2.4. Selection Process

Records exported from each database (.ris for Scopus; .csv for Web of Science; .nbib for ERIC) were merged into a single file that included a source-database field for audit purposes, using a custom, version-controlled Python 3.14.6 script that performed an exact DOI match followed by a fuzzy match (normalized title + year + first author’s surname; similarity threshold ≥ 0.90), with every match logged. Raw counts before deduplication were as follows: Scopus (n = 92), Web of Science (n = 123), and ERIC (n = 199), for a total of 414 records. Deduplication was carried out in two sequential stages: merging Scopus (n = 92) with Web of Science (n = 123) removed 39 duplicates, leaving 176 unique records; this set, merged with ERIC (n = 199), removed a further 34 duplicates, leaving 341 unique records that proceeded to title/abstract screening.

Title and abstract screening (Phase 1) was conducted independently by two reviewers (L.I.P.S. [R1] and M.R.S.C. [R2]) based on the eligibility criteria, using a structured, validated screening worksheet that included a standardized catalog of exclusion codes and an automated concordance detection system. Specifically, an AI-based language model (Claude, Anthropic) was used to generate an initial eligibility determination for each title and abstract according to the E1–E7 catalog; each AI-generated determination was then independently re-evaluated by the same reviewers (R1, R2). Following this verification process, agreement between the human raters was almost perfect: Cohen’s κ = 0.836 (95% CI [0.770, 0.902]; observed agreement = 93.5%), per the Landis and Koch (1977) benchmark criteria, exceeding the a priori threshold of κ ≥ 0.60 set in the protocol. Of the 341 records assessed, 22 (6.5%) presented discrepancies that were resolved through arbitration (J.D.D.C.); the arbiter’s decision agreed with R1 in 19 cases (86.4%) and with R2 in 3 cases (13.6%). Following arbitration, 89 records were included and proceeded to full-text assessment (Phase 2); 252 were excluded.

**Figure 1:**
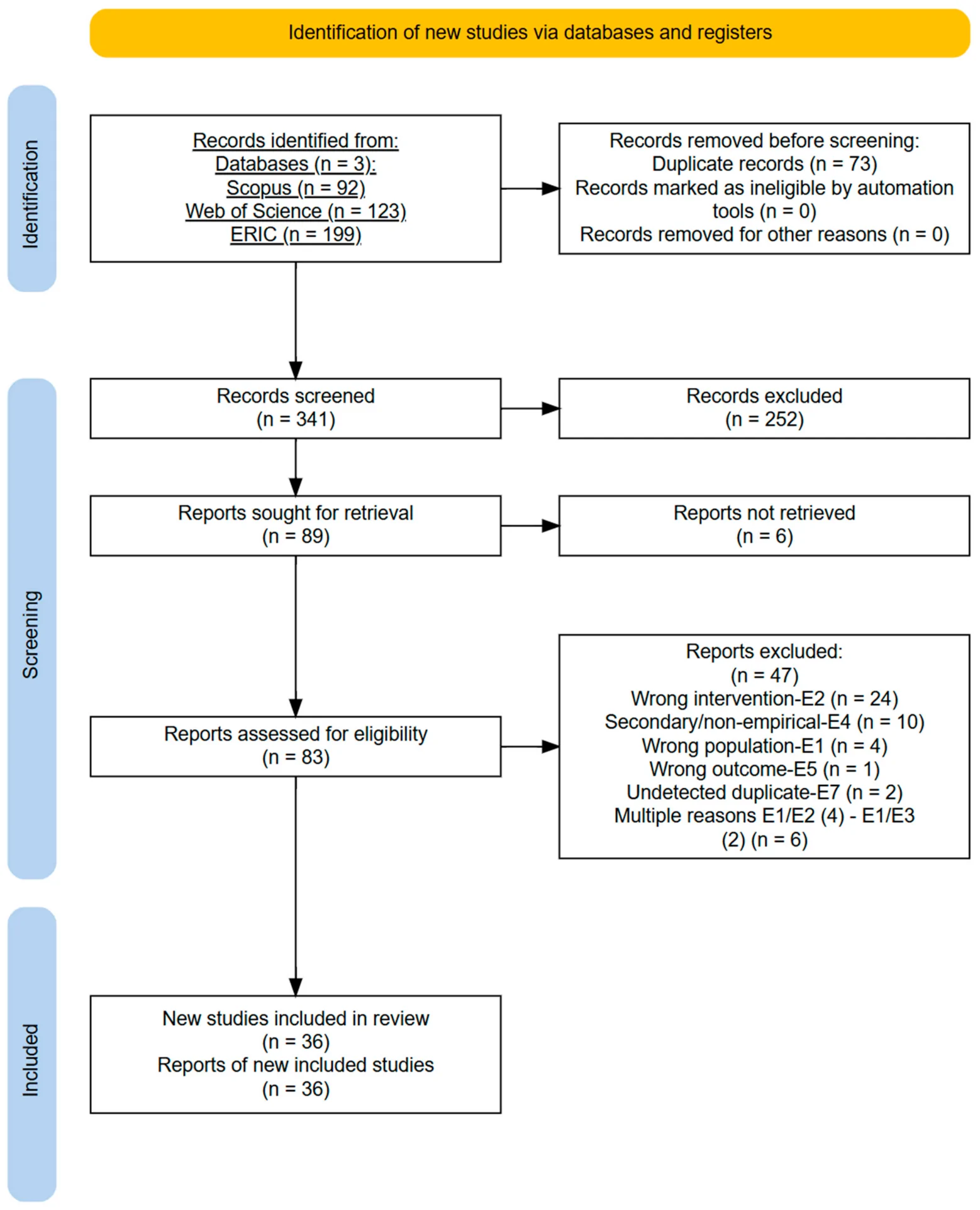
PRISMA 2020 flow diagram for the study selection process (Page et al., 2021). Note. Diagram adapted from “The PRISMA 2020 statement: An updated guideline for reporting systematic reviews,” by M. J. Page et al., 2021, BMJ, 372, n71 (https://doi.org/10.1136/bmj.n71). CC BY 4.0.

In Phase 2, full texts were sought for 89 records; 6 could not be retrieved despite an exhaustive search (institutional access, interlibrary loan, and author contact) and were excluded under code E7. Two reviewers (R1, R2), working independently, assessed the eligibility of the remaining 83 reports based on their full text, using criteria E1–E7. Of 20 discrepancies, including four involving records reclassified from an initial judgment favoring inclusion after an exclusion feature was identified only upon full-text examination, all were resolved by consensus or, when consensus could not be reached, through arbitration (J.D.D.C.). Of the 83 reports assessed, 47 were excluded (incorrect intervention, E2, n = 24; secondary or non-empirical research, E4, n = 10; incorrect population, E1, n = 4; incorrect outcome, E5, n = 1; duplicates undetected at the time of assessment, E7, n = 2; six reports met more than one exclusion criterion: four E1/E2 and two E1/E3), leaving 36 studies included in the review. Data extraction and methodological quality appraisal of the 36 included studies were performed independently by both reviewers; Cohen’s κ coefficients for inter-rater reliability were 0.973 (95% CI [0.961, 0.985]) for data extraction and 0.977 (95% CI [0.964, 0.989]) for MMAT appraisal, indicating almost perfect agreement in both cases (Landis & Koch, 1977). The PRISMA 2020 flow diagram is shown in Figure 1.

Of these 20 arbitrated conflicts, 14 records had appeared, on initial independent screening, to meet the eligibility criteria but were ultimately excluded after closer full-text examination; these borderline records are cited individually, with their final exclusion code and rationale, in Supplementary Table S9.

### 2.5. Data Extraction

A structured data extraction form, previously validated through a pilot study involving five studies, was used to extract the variables listed in Table 3 from the 36 included articles. As in the Phase 2 selection process, two reviewers (L.I.P.S. and M.R.S.C.) independently assessed each study to extract and verify every value; discrepancies were resolved by consensus. Active coaching components (modeling, guided practice, reflection, observation, goal setting, and performance feedback) were coded as binary indicators (yes/no) according to the codebook’s explicit definitions. Assigning these codes required direct textual evidence that the component occurred within the actual coach–teacher interaction, rather than during the preceding group training session. The six components were coded using predefined operational definitions specified in the project codebook (OSF M3846) before data extraction began, and were not mutually exclusive: a single study could be coded “yes” on any number of the six components simultaneously if each met its own definition independently, since most Practice-Based Coaching-derived models combine several components by design (Section 3.3.2). Overlap between conceptually adjacent components (e.g., distinguishing “modeling” from “guided practice,” or “reflection” from “performance feedback”) was resolved by the codebook’s requirement that each component be coded only when the study text described it as a discrete, identifiable activity, rather than inferred from the general label a study gave its own coaching model.

Missing or unclear information was handled using a standardized convention applied consistently across the data extraction matrix (Section 2.8). A field was coded “N/A” when a variable did not apply to a study by design (e.g., a reflexivity statement for a purely quantitative study, or an effect-magnitude statistic for a qualitative study), and coded “Not reported in the reviewed excerpt” when the variable was structurally applicable but the published text did not provide enough information to extract it (e.g., a funding statement absent from the available excerpt). No summary-statistic conversions or imputations were performed on any extracted variable, consistent with the decision not to pool effect sizes across studies (Section 2.7.2); each study’s fidelity, dosage, quality, and outcome information is reported in its own original metric in Table 4 and Supplementary Table S4. Discrepancies in how a variable should be coded, including whether it was missing or inapplicable, were resolved by consensus between the two independent extractors (Section 2.5, para 1).

### 2.6. Methodological Quality Appraisal

The methodological quality of the included studies was appraised using the 2018 Mixed Methods Appraisal Tool (MMAT; Hong et al., 2018). This tool includes two screening questions and five sets of criteria specific to each study design (qualitative, randomized controlled trial, non-randomized quantitative, descriptive quantitative, and mixed methods), each rated exclusively as “yes,” “no,” or “cannot tell.” Following the expansion of eligible study designs in protocol version 1.7, the five MMAT categories were applied to the 36 included studies, which were classified and appraised as follows: 5 exclusively qualitative studies, 9 randomized controlled trials, 7 non-randomized quantitative studies, 5 descriptive quantitative studies, and 10 mixed-methods studies (7 combining a qualitative and a descriptive quantitative component; 3 combining a qualitative and a non-randomized quantitative component).

Item-level responses are reported individually and are not synthesized into an overall score, in strict compliance with the methodological guidance established by the instrument’s developers (Hong et al., 2018). The verification process for each study was carried out independently by two reviewers (A.P.C. and C.V.C.C.), and detected discrepancies were resolved by consensus. Finally, methodological quality was not used as an exclusion criterion; instead, this factor was explicitly incorporated into the interpretation of findings, including a post hoc sensitivity analysis restricted to the subset at lowest risk of bias (Section 3.2).

Beyond assessing risk of bias within individual studies, we examined risk of bias due to missing results at the level of the synthesis (Page et al., 2021, item 14). For each of the 36 included studies, data extraction recorded whether implementation fidelity, dosage, quality, and student-outcome indicators were reported in the available text (Table 3, “Outcomes” domain). Because none of the included studies were traceable to a publicly registered protocol or trial-registry record that could be checked against the published report on an outcome-by-outcome basis, a formal registered-versus-reported-outcome comparison was not feasible. Instead, the completeness with which each implementation and outcome indicator was reported across the corpus was tabulated as a synthesis-level proxy for missingness (Section 3.2), interpreted narratively alongside the possibility that non-reporting reflects a study’s original scope (e.g., a process-focused study that did not set out to measure student outcomes) rather than selective suppression of unfavorable results.

### 2.7. Data Synthesis

Following the expansion of eligible designs introduced in version 1.7, this review adopts a convergent segregated mixed-methods synthesis design, in accordance with JBI methodological guidance for mixed-methods systematic reviews (Stern et al., 2020). Qualitative and quantitative evidence are synthesized separately, using methods appropriate to each data type, and both syntheses are subsequently integrated in a final configuring stage. This design was chosen over the convergent integrated approach (which requires transforming one data type into another) because the review question addresses two distinct but complementary dimensions: the qualitative experience and mechanisms of coaching, and the quantitative magnitude of its effects on implementation and student outcomes.

#### 2.7.1. Qualitative Component

Thematic synthesis (Thomas & Harden, 2008) is applied to all qualitative data, drawn from qualitative studies and the qualitative components of mixed-methods studies (15 of the 36 included studies), in three phases: (1) line-by-line coding of the textual content of the “Results” and “Discussion” sections, using qualitative analysis software (ATLAS.ti, version 23 or later); (2) inductive development of descriptive themes that remain faithful to the main findings of the studies; (3) generation of analytical themes through second-order interpretation, organized around the components of active coaching most consistently associated with improvements in implementation, contextual facilitators and barriers, hypothetical mechanisms linking teacher change to student outcomes, and conditions for sustainability. As a concrete illustration of this progression, first-order descriptive codes such as “coach observes lesson,” “written feedback after observation,” and “teacher sets a specific practice goal” were grouped inductively into the descriptive theme “observation-feedback-goal cycle,” which was then interpreted, alongside descriptive codes on trust and non-evaluative framing, into the second-order analytical theme reported as Macro-theme 2 (Section 3.3.2). This example is illustrative rather than a full audit trail: the underlying first-order coding records are archived in the project’s ATLAS.ti file (OSF M3846) rather than reproduced as a supplementary table, since they comprise several hundred codes across the 15 qualitative-contributing studies. Supplementary Table S7 instead reports the GRADE-CERQual assessment of the resulting review findings (contributing studies, methodological limitations, coherence, adequacy of data, relevance, and overall confidence), which presents the products of this coding process rather than the coding trail itself. The quality of the presentation of the qualitative findings follows the ENTREQ guidelines (Tong et al., 2012), and the certainty of each finding in the qualitative review is assessed using GRADE-CERQual (Lewin et al., 2018; methodological limitations, coherence, adequacy of data, and relevance), reported in the Summary of Qualitative Findings table (Table S7 of the Supplementary Materials).

#### 2.7.2. Quantitative Component

Data from the quantitative studies and from the quantitative components of the mixed-methods studies (31 of the 36 included studies), namely effect estimates, fidelity/dosage statistics, and significance tests, are presented in tables and summarized narratively by active coaching component and outcome type, following the Synthesis Without Meta-analysis (SWiM) reporting guidelines (Campbell et al., 2020) where applicable. No meta-analysis (statistical pooling of effect sizes) was performed, for three reasons: (i) the conceptual and operational heterogeneity across coaching components, fidelity instruments, comparison conditions, and outcome measures throughout the expanded evidence base exceeds reasonable homogeneity assumptions for pooling; (ii) the review’s objective remains interpretive-explanatory and centers on the qualitative strand, with the quantitative strand playing a complementary, configuring role; (iii) the segregated convergent design (Stern et al., 2020) explicitly accommodates narrative quantitative synthesis as an alternative to meta-analysis when heterogeneity precludes pooling. We specifically considered whether a more homogeneous subset, such as the nine randomized controlled trials, might support limited pooling; however, even within this subset, studies used non-overlapping outcome instruments and constructs (e.g., the parent/teacher-rated Strengths and Difficulties Questionnaire in Berry et al. (2016), versus a battery of social-emotional, behavioral, and developmental child measures in Hemmeter et al. (2021), versus direct classroom observation of teacher practice and child behavior in Morris et al. (2013); full instrumentation for each study is reported in Supplementary Table S4) and different comparison conditions (business-as-usual versus active alternative training), so no subset was judged sufficiently homogeneous in outcome operationalization to support even a restricted meta-analysis. Methodological quality (Section 2.6) and consistency in effect direction across studies are analyzed narratively rather than formally rated.

Possible causes of heterogeneity among the quantitative results were explored narratively, in place of the subgroup analysis or meta-regression methods used in meta-analytic reviews: the direction and consistency of findings (Supplementary Table S4) were cross-tabulated against candidate moderating characteristics recorded during data extraction (Table 3): delivery modality, coach affiliation (internal/external), study-design rigor, and geography, with results reported narratively in Section 3.2.

To complement the GRADE-CERQual assessment of the qualitative strand (Section 2.7.1), certainty in five key quantitative findings was appraised using a simplified adaptation of the GRADE domains (risk of bias, inconsistency, indirectness, imprecision, and reporting bias; Guyatt et al., 2011), adapted for narrative synthesis without meta-analysis. Ratings were informed by the MMAT appraisal (Section 2.6; Supplementary Table S8), the direction-of-effect concordance described in Section 3.3.4, and the reporting-completeness analysis in Section 3.2. Each domain was rated by consensus among the reviewer team as not serious, serious, or very serious, yielding an overall certainty rating (High, Moderate, Low, or Very low) for each finding, reported in Supplementary Table S10 and summarized in Section 3.2.

#### 2.7.3. Integration

The qualitative and quantitative syntheses are integrated into a final narrative configuration, in which qualitative themes are juxtaposed with the pattern of quantitative results (for example, whether the components qualitatively identified as salient correspond to the components showing a consistent quantitative effect), presented in a joint table (rows: active components/themes; columns: qualitative findings, quantitative findings, integrated interpretation).

### 2.8. Ethical Approval and Data Availability

This study is a systematic review of previously published, publicly available literature; it did not involve the collection of new data from human or animal participants, and ethical approval was therefore not required. Upon completion of the review, the following materials will be deposited in the OSF repository associated with the registered protocol (project M3846): the complete Phase 1 and Phase 2 screening-and-extraction workbooks (title/abstract and full-text screening decisions with exclusion codes and arbitration records; the data extraction matrix; and the item-level MMAT 2018 appraisals), the custom deduplication Python script referenced in Section 2.4, original bibliographic export files, ATLAS.ti coding files, the completed PRISMA 2020 checklist, and the full protocol version history with its amendment log.

### 2.9. Use of Generative Artificial Intelligence

In conducting this review, a generative artificial intelligence model (Claude, Anthropic) was used as an instrumental and analytical tool across several methodological phases. During Phase 1 (title and abstract screening), the tool assisted with the preliminary structuring of eligibility criteria, optimization of the literature search, and translation of records in languages other than English or Spanish, while also supporting preliminary data extraction for audit purposes. Artificial intelligence was not used to generate primary data from the studies, to draw substantive research conclusions, or to substitute for the independent judgment of the designated reviewers. Rather, automated outputs were treated strictly as drafts requiring human verification at every stage, as documented in the project workbook’s audit trail. No AI-generated eligibility determination was accepted without independent human re-evaluation: as detailed in Section 2.4, every one of the 341 title/abstract determinations and every one of the 83 full-text determinations was independently re-assessed by both human reviewers (R1, R2) against the E1–E7 criteria, blind to the AI-generated determination where the workflow allowed it, and any disagreement between a reviewer’s judgment and the AI-generated determination was resolved using the same consensus/arbitration procedure applied to human-to-human disagreements (J.D.D.C.). The AI-assisted preliminary data extraction (Section 2.5) was subject to the identical two-reviewer independent verification and consensus process as manually extracted data, with no distinction in the level of scrutiny applied.

## 3. Results

### 3.1. General Corpus Characteristics and Methodological Configuration

Screening, documented in the PRISMA 2020 flow diagram (Figure 1), yielded a final corpus of 36 primary studies. Populations were predominantly in-service early-childhood and elementary teachers, with fewer studies focused on secondary education. Sample sizes varied enormously: from qualitative and single-case designs with fewer than 15 teachers to cluster-randomized trials with more than 100 educators across multiple school districts. Details of each intervention (teacher demographics, duration, active components) are consolidated in Table 4.

Following PRISMA 2020 item 19, Supplementary Table S4 presents, for each study, its reported effect estimate together with whatever precision statistic the primary study itself provided (a confidence interval, standard deviation, test statistic, or *p*-value). A single, uniform precision metric could not be reported across the corpus because primary study designs range from cluster-randomized trials reporting confidence intervals (e.g., Berry et al., 2016) to single-case designs using visual analysis or non-overlap indices such as TAU-U or PND (e.g., Robbie, 2021; Rakap & Balikci, 2025), for which conventional confidence intervals are not computed; each study’s precision statistic is therefore retained in its own original metric rather than converted to a common format.

Geographically, the evidence is concentrated in North America, with occasional contributions from Europe (United Kingdom: Berry et al., 2016; Norway: Skåland et al., 2023; Turkey: Rakap & Balikci, 2025) and a still-incipient presence from Latin America and Asia. This concentration, revisited in the Discussion as an external-validity limitation (Section 4.2), coincides with a shift in the type of design employed: the field has moved from small-scale efficacy trials (single-case or multiple-baseline) testing the feasibility of active ingredients (Fox et al., 2011; Stoiber et al., 2022) toward large-scale cluster-randomized effectiveness trials (Bottiani et al., 2026; Hemmeter et al., 2021). The studies sit at the intersection of educational psychology, implementation science, and continuing teacher education, and share an interest in measuring how trained skills transfer from the workshop to everyday classroom practice.

Given this plurality of designs, a convergent segregated mixed-methods synthesis was adopted (JBI; Stern et al., 2020): qualitative and quantitative findings were first analyzed separately and then integrated in the interpretive phase presented below. The qualitative component also followed the ENTREQ framework (Tong et al., 2012), which helped trace the path from each study’s first-order findings to the second- and third-order interpretive constructs: the macro-themes of Section 3.3.

### 3.2. Methodological Quality of the Evidence

Appraisal with the Mixed Methods Appraisal Tool (MMAT; Hong et al., 2018) places the corpus at a moderate-to-high level of evidence, with systemic vulnerabilities characteristic of research conducted in real school ecologies. Its most consistent strength is fidelity measurement: the highest-quality studies no longer rely solely on teacher self-report but on validated, independent observational coding, such as the Teaching Pyramid Observation Tool (TPOT) or the Classroom Assessment Scoring System (CLASS), which reduces common-method variance. The extensive use of Hierarchical Linear Models also enabled precise modeling of variance nested across students, classrooms, and schools.

Risk of bias by synthesis (macro-theme). Macro-theme 1 (input ecology; Section 3.3.1) draws primarily on descriptive-quantitative and mixed-methods studies (e.g., Cramer et al., 2021; Hunter et al., 2022; Harrington, 2024), with a smaller qualitative contribution (Wanless et al., 2013); its central vulnerability is the correlational, non-experimental nature of most contributing designs, which limits causal claims about which systemic or individual conditions drive fidelity. Macro-theme 2 (active architecture and consultative alliance; Section 3.3.2) draws on single-case and quasi-experimental studies with comparatively strong internal validity for the coaching-fidelity link (Fox et al., 2011; Hemmeter et al., 2015; Rakap & Balikci, 2025) and one RCT (J. A. Welsh et al., 2024), but its claims about relational or alliance quality specifically rely more heavily on correlational and qualitative evidence (Hardy et al., 2025), for which common-method variance is a risk. Macro-theme 3 (scaling tensions and equity; Section 3.3.3) rests on the smallest and most heterogeneous evidence base in the corpus: one cluster-RCT (Bottiani et al., 2026) alongside predominantly qualitative and mixed-methods studies (Arias de Sanchez & Li, 2025; Partee et al., 2023), consistent with the caution already expressed for this theme in the sensitivity analysis above. Macro-theme 4 (translational cascade; Section 3.3.4) draws disproportionately on the corpus’s strongest designs, including four RCTs (Berry et al., 2016; Hemmeter et al., 2021; Morris et al., 2013; Downer et al., 2018), which is precisely why the null-to-marginal academic-outcome pattern reported there carries more methodological weight than a similarly null finding would in a qualitative-dominated theme.

Two risks of bias recur throughout the corpus, however. Blinding is virtually unfeasible in behavioral interventions, leaving room for the Hawthorne effect and social desirability, particularly when the same consultant who coaches the teacher also evaluates them. Differential attrition, compounded by teacher turnover, occupational burnout, and, in several studies, the pandemic (Garcia et al., 2022; Robbie, 2021), compromises the ecological validity of the larger-scale longitudinal designs. A recurring pattern is also apparent: university-led coaching models sustain treatment integrity better than decentralized peer-coaching models, suggesting that much of the reported methodological quality reflects support conditions that are difficult to replicate outside the research context.

Reporting completeness across the corpus. Of the 36 included studies, implementation fidelity was reported for 27 (75.0%), dosage for 23 (63.9%), an independent quality or competence rating for 22 (61.1%), and a student-level outcome for 15 (41.7%) (Supplementary Tables S3 and S4). This declining gradient, from process indicators, most consistently reported, to distal student outcomes, least consistently reported, is consistent with the translational-cascade pattern described in Macro-theme 4 (Section 3.3.4). We interpret this pattern with caution: for several studies, the absence of a given indicator reflects a deliberate scope restriction (e.g., an implementation-process report whose child outcomes are published in a companion report, as in Mattera et al., 2013) rather than evidence of selective outcome suppression. No systematic pattern was found in which a study’s introduction or methods promised a specific fidelity, dosage, or outcome measure that was subsequently absent from its results section, which would be the clearer signature of within-study selective reporting.

Investigation of heterogeneity among quantitative results. Because no meta-analysis was performed, heterogeneity was explored narratively by cross-tabulating the direction and consistency of quantitative findings (Supplementary Table S4) against candidate moderating characteristics recorded during data extraction (Table 3): delivery modality, coach affiliation (internal/external), study-design rigor, and geography. Three patterns emerged. First, delivery modality: fully asynchronous, video-mediated coaching formats (J. A. Welsh et al., 2024; J. Welsh et al., 2025) were the only studies in the corpus to report declines in teacher adherence or engagement, whereas in-person and blended video-plus-live-feedback formats (e.g., Downer et al., 2018; Siller et al., 2024; Spacciapoli et al., 2022) did not report this pattern, suggesting modality, rather than active-ingredient content, as a plausible moderator of the relational-dilution effect described in Macro-theme 2 (Section 3.3.2). Second, coach affiliation: internal/peer-coaching studies (Giordano et al., 2021; Robbie, 2021) reported mixed adherence findings relative to external-coach studies, consistent with the trade-off discussed in Section 3.3.2 rather than a uniform advantage for either model. Third, outcome type and reporter: divergence in academic-outcome findings tracked closely with whether the outcome was teacher-self-reported (positive; e.g., Doyle et al., 2023) or independently or standardized-assessed (null or marginal; e.g., Berry et al., 2016; Hemmeter et al., 2021; Morris et al., 2013), the pattern already introduced in Macro-theme 4 (Section 3.3.4), rather than with geography or study-design rigor, neither of which showed a consistent association with effect direction in this corpus.

Sensitivity analysis of the qualitative synthesis. Following PRISMA 2020 item 20d (Page et al., 2021), we tested whether the macro-themes in Section 3.3 held when the analysis was restricted to the studies at lowest risk of bias, defined as those rated “Yes” on at least four of the five applicable MMAT criteria (Bottiani et al., 2026; Hemmeter et al., 2015, 2021; Fox et al., 2011; Rakap & Balikci, 2025; Wanless et al., 2013). Consensus on the centrality of the non-evaluative consultative alliance and on the superiority of active ingredients over traditional training remained stable within that subset, ruling out that these are artifacts of weaker designs. The conclusion regarding the differential transfer of peer coaching, by contrast, rests on fewer high-rigor studies and should be read with greater caution.

Certainty of the quantitative evidence. Applying the framework described in Section 2.7.2 to five key quantitative findings (Supplementary Table S10) yielded moderate certainty for the core finding that structured, active-ingredient coaching cycles improve teacher implementation fidelity relative to training-only conditions, and moderate certainty that short-term effects on independently assessed academic outcomes are null or marginal. Certainty was low for the differential effectiveness of internal versus external coaching and for the equity-focused R-CITY supplement findings (Bottiani et al., 2026), both resting on a small number of studies, and low-to-very-low for claims about asynchronous video-coaching engagement, which rest on only two studies from the same research group (J. A. Welsh et al., 2024; J. Welsh et al., 2025).

### 3.3. Thematic Synthesis of the Evidence

**Figure 2:**
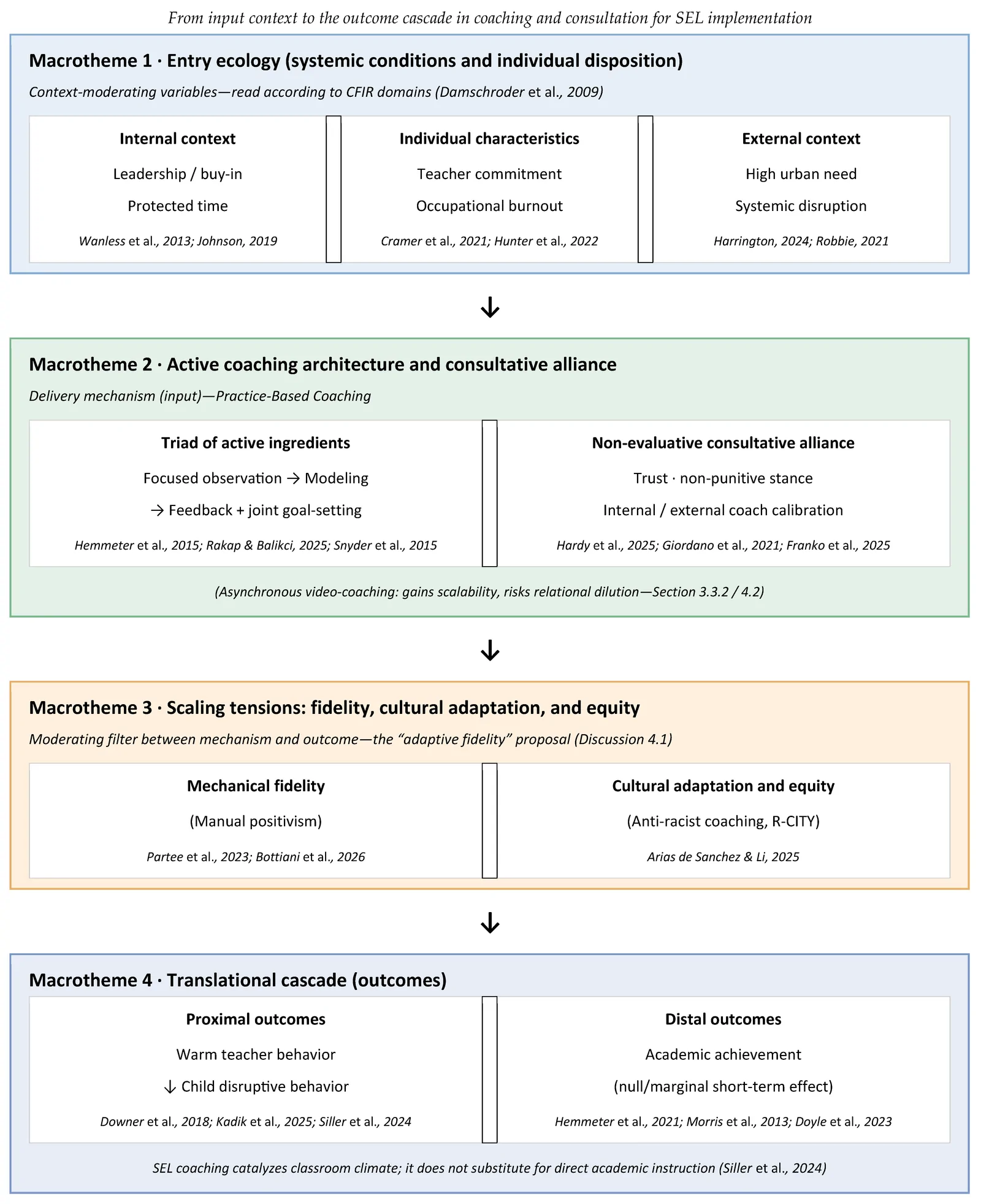

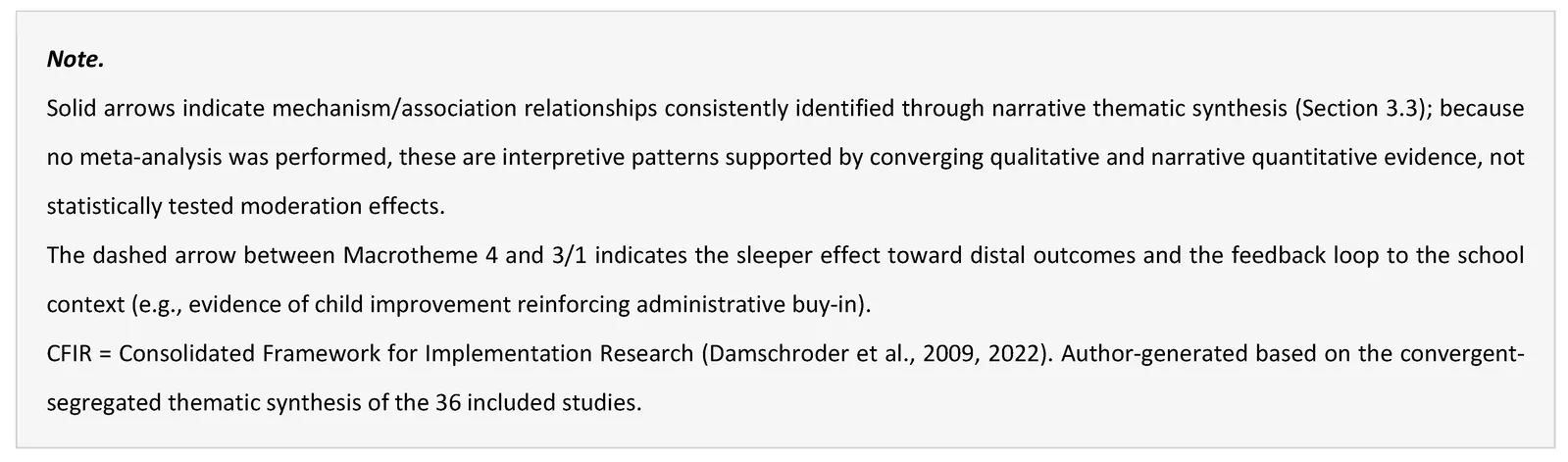
Logic Model of the Thematic Synthesis. Sources: (Arias de Sanchez & Li, 2025; Bottiani et al., 2026; Cramer et al., 2021; Damschroder et al., 2009, 2022; Downer et al., 2018; Doyle et al., 2023; Franko et al., 2025; Giordano et al., 2021; Hardy et al., 2025; Harrington, 2024; Hemmeter et al., 2015, 2021; Hunter et al., 2022; Johnson, 2019; Kadik et al., 2025; Morris et al., 2013; Partee et al., 2023; Rakap & Balikci, 2025; Robbie, 2021; Siller et al., 2024; Snyder et al., 2015; Wanless et al., 2013).

Cross-cutting analysis of the corpus yielded four interdependent macro-themes that explain the mechanisms, context, and outcomes of teacher coaching and consultation for SEL implementation. Figure 2 summarizes these relationships as an input–process–outcome logic model.

#### 3.3.1. Macro-Theme 1: The Input Ecology—Systemic Conditions and Individual Disposition

Coaching’s success does not depend solely on its technical design but on the systemic conditions and individual disposition that frame the school. Using the domains of the Consolidated Framework for Implementation Research (CFIR; Damschroder et al., 2009, 2022) to organize the evidence, within the inner setting, school-leadership support, particularly administrative commitment, stands out as the primary regulator of fidelity (Wanless et al., 2013; Johnson, 2019): teachers identify the lack of protected time and logistical resources as the most frequent structural barrier, and perceive coaching as legitimate support only when administration aligns SEL with its priorities. At the level of individual characteristics, Cramer et al. (2021) show that educators’ own commitment to implement predicts delivery dosage and quality throughout the year, although that disposition can be eclipsed by occupational burnout, the accumulation of pedagogical initiatives, and the salary tensions typical of early-childhood education (Hunter et al., 2022). Within the outer setting, high-need urban environments or periods of systemic disruption, including the pandemic, appear to absorb the cognitive bandwidth that teachers would need to assimilate coaching (Harrington, 2024; Robbie, 2021).

From this convergence emerges a function that the coach ends up assuming almost out of necessity: shifting, at least temporarily, from technical-pedagogical assistance to providing socioemotional containment for the teacher themselves, as a prerequisite for being able to influence curricular fidelity (Partee et al., 2023; Jennings et al., 2017).

#### 3.3.2. Macro-Theme 2: The Active Architecture of Coaching and the Consultative Alliance

Effective coaching combines two ingredients that do not function in isolation: a technical architecture and a relational alliance that legitimizes its reception. Training models reduced to initial workshops are insufficient to translate declarative knowledge into sustained practice (Fox et al., 2011; Hemmeter et al., 2015; Stoiber et al., 2022); instead, an iterative cycle of data-based observation, in-context modeling, and feedback with joint goal setting (action planning) is consistently associated with greater implementation fidelity (Hemmeter et al., 2015; Rakap & Balikci, 2025; Snyder et al., 2015), as documented by the Practice-Based Coaching (PBC) framework.

Scaling this architecture introduces a tension inherent to technological mediation. Asynchronous video-coaching allows teacher micro-interactions to be broken down without the logistical constraints of in-person observation, and generates powerful moments of self-discovery when teachers observe themselves (Downer et al., 2018; Siller et al., 2024; Skåland et al., 2023; Spacciapoli et al., 2022). But that same efficiency can run up against psychosocial barriers: purely virtual formats are associated with declines in teacher adherence and engagement (J. A. Welsh et al., 2024; J. Welsh et al., 2025), as if asynchrony optimized dosage at the cost of diluting the bond, turning a space intended for reflection into just another administrative task added to teachers’ workload.

That relational dilution connects to the second ingredient: technical components yield little, or are even counterproductive, if they are not sustained by a consultative alliance grounded in trust, mutual respect, and an explicitly non-evaluative stance (Arias de Sanchez & Li, 2025; Bottiani et al., 2026; Franko et al., 2025). Hardy et al. (2025), through the Coaching Quality (CQC) framework, show that empathic listening and support are a necessary condition for directive feedback to actually modify classroom socioemotional practice; when coaching is perceived as auditing, active ingredients fail and superficial compliance emerges.

Regarding who should deliver coaching, the evidence is less clear-cut than it first appears. Internal coaching capitalizes on institutional trust and may marginally favor adherence and child outcomes (Giordano et al., 2021), but exposes peer-coaches to a role conflict (being seen as an extension of administration) and may lack the technical rigor of an experienced external consultant (Robbie, 2021). External coaching faces more initial friction in building rapport, though it guarantees clearer non-evaluative boundaries (Franko et al., 2025). This finding rests on fewer high-rigor studies (Section 3.2) and should therefore be read as a trade-off of strengths rather than the superiority of one model over another: what appears decisive is calibrating relational support with technical precision, ensuring that coaching is never perceived as punitive evaluation.

#### 3.3.3. Macro-Theme 3: Scaling Tensions—Fidelity, Cultural Adaptation, and Equity

When evidence-based SEL programs leave the controlled trial and scale up into real educational systems, friction emerges between fidelity to the manual and the need to adapt the intervention to culturally heterogeneous classrooms and child-centered pedagogies (Arias de Sanchez & Li, 2025). Excessive rigidity erodes teacher commitment, which responds with spontaneous modifications, omissions, or substitutions, whether due to time constraints or a mismatch with their students (J. Welsh et al., 2025).

Equity is a particularly sensitive axis of this tension. Partee et al. (2023) warn that mental-health consultation and traditional SEL can unintentionally reproduce “deficit” frameworks that convey to families and educators the idea that children from marginalized backgrounds must be “corrected” to fit behavioral norms of Eurocentric origin. Against this assimilationist approach, Bottiani et al. (2026) show that incorporating an equity supplement (R-CITY) and coaching focused on implicit bias is associated with less hostile attribution bias and greater teacher self-efficacy with diverse populations.

We develop this pattern as a theoretical contribution in the Discussion (Section 4.1), but its core is worth anticipating here: contemporary coaching appears to need to move away from the role of “compliance monitor” and become a mediator of adaptations that safeguard SEL’s active ingredients without demanding cultural homogeneity.

#### 3.3.4. Macro-Theme 4: The Translational Cascade—From Teacher Behavior to Student Impact

Coaching’s impact follows a translational cascade: the intervention first modifies the educator’s proximal practice, and that modification is the mechanism that subsequently affects student development. There is robust empirical support that high-fidelity coaching is associated with warmer teacher interactions, greater emotional scaffolding, and less disruptive and aggressive classroom behavior (Downer et al., 2018; Kadik et al., 2025; Siller et al., 2024), with a consequent reduction in exclusionary disciplinary practices.

Where consensus breaks down is in the transfer of these effects to academic performance. The divergence appears to depend largely on who reports the outcome and over what time distance: teacher self-report tends to show academic improvements paralleling coaching (Doyle et al., 2023), whereas large-scale trials using standardized, independent cognitive assessments detect, almost without exception, null or marginal short-term impacts on early literacy or mathematics (Hemmeter et al., 2021; Morris et al., 2013).

This does not invalidate the intervention: rather, it suggests that SEL coaching directly affects the classroom’s micro-architecture and children’s self-regulation, and that its translation into distal academic outcomes would be sequential rather than immediate: a “sleeper effect” in which a stabilized social climate would scaffold a cognitive maturation process that exceeds the typical 6-to-12-month follow-up windows of most current quasi-experimental designs (Doyle et al., 2023). Socioemotional coaching would thus act as a catalyst for classroom climate, not as a substitute for high-quality direct academic instruction (Siller et al., 2024).

### 3.4. Integration of Qualitative and Quantitative Evidence

Table 5 operationalizes the integration procedure described in Section 2.7.3, juxtaposing, for each macro-theme, the qualitative pattern identified in Section 3.3 against the corresponding quantitative evidence, and stating whether the two strands converge, partially converge, or diverge.

## 4. Discussion

Before turning to the conceptual and practical implications of these findings, we briefly summarize the core pattern established in the Results: coaching and consultation are associated with the most consistent gains in SEL implementation fidelity when they combine an active technical architecture (data-based observation, modeling, and feedback tied to jointly set goals; Section 3.3.2) with a non-evaluative, trust-based consultative alliance (Section 3.3.2), under school conditions that provide protected time and leadership support (Section 3.3.1). Effects on proximal teacher practice and classroom climate are well supported (Section 3.3.4), whereas short-term effects on independently assessed academic outcomes are consistently null to marginal, and effects vary by fidelity-adaptation trade-offs when programs scale across cultural contexts (Section 3.3.3). The sections below interpret this pattern theoretically (Section 4.1), geographically (Section 4.2), and in terms of policy and practice (Section 4.3), before turning to the limitations of the review (Section 4.4).

### 4.1. Toward a New Conceptual Framework: Adaptive Fidelity

The friction identified in Macro-theme 3 between mechanical fidelity and cultural responsiveness (Section 3.3.3) is not merely an implementation limitation: it is also the starting point for a conceptual proposal. We propose calling adaptive fidelity the coaching model in which the twenty-first-century consultant no longer monitors mechanical compliance with a manual, a positivist stance rooted in standardization, but instead safeguards the program’s active ingredients (observation, modeling, feedback, goal setting; Section 3.3.2) while fostering cultural and community responsiveness.

Under this framework, adaptive fidelity is not a midpoint between compliance and adaptation, but a distinct function: that of a mediator who distinguishes between a program’s core elements, whose alteration compromises its causal mechanism, and its surface elements, whose flexibility is indispensable to the intervention’s cultural legitimacy. Bottiani et al.’s (2026) findings on the R-CITY supplement, read alongside the tensions Partee et al. (2023) describe regarding deficit frameworks, empirically illustrate this distinction: incorporating an explicit implicit-bias component does not dilute coaching’s active ingredients but rather broadens their relational reach without sacrificing their technical architecture. This concept speaks directly to this Special Issue’s agenda on teacher training and multicultural perspectives. To be transparent about its evidentiary status: the tension between mechanical fidelity and cultural responsiveness, and the specific empirical illustrations from Bottiani et al. (2026) and Partee et al. (2023), are grounded directly in the thematic synthesis (Section 3.3.3). The label “adaptive fidelity,” its formulation as a distinction between a program’s core and surface elements, and its framing as a shift in the coach’s role, are a higher-order theoretical interpretation proposed by the review team, not a construct that any single included study names or tests directly; we present it as a conceptual contribution for the field to evaluate, rather than as an empirical finding of the review.

### 4.2. Geographic Representativeness and the Global South Gap

As anticipated in Section 3.1, the reviewed corpus is dominated by U.S.-based research, with occasional contributions from Europe (Berry et al., 2016; Skåland et al., 2023) and a still-marginal presence from Latin America, Africa, and Asia. This geographic concentration is not merely an artifact of the databases consulted: it constrains the external validity of the reviewed models, nearly all of which were designed for resource conditions (moderate teacher–student ratios, dedicated consultation staff, stable technological infrastructure) that are far from universal.

The barriers characteristic of lower-resource contexts (scarce specialized staff, high student–teacher ratios, common across much of Latin America and other Global South regions) call for coaching models less intensive in staffing and logistics than traditional in-person protocols. The hybrid video-coaching models identified in this corpus (Spacciapoli et al., 2022; Siller et al., 2024) point in that direction: by decoupling technical feedback from a consultant’s continuous physical presence, they allow the active ingredients of Macro-theme 2 to be preserved with fewer specialized staff per school. As discussed in that same section, however, the design must ensure that asynchrony does not dilute the bond of the consultative alliance, especially where teacher overload is already high. It remains for future research to evaluate these hybrid models in low-resource schools outside North America, and to test whether adaptive fidelity (Section 4.1) holds up with less technical support.

### 4.3. Implications for Policy and Practice

Macro-theme 1 (Section 3.3.1), on the role of the school’s inner setting and protected time as regulators of fidelity, carries a direct policy implication that the scale-up literature addresses too little: investing in SEL programs without simultaneously allocating protected time for the coaching observation–feedback cycle likely yields less than expected. A program that is “well designed” on paper but lacks the systemic conditions of Macro-theme 1 risks ending up in the superficial compliance described in Macro-theme 2 (Section 3.3.2).

In actionable terms for school leaders and policymakers:

1. Funding for SEL curricula should be conditioned on protected hours that are contractually safeguarded rather than merely available, so that teachers can receive the full observation-and-feedback cycle from a coach; without that structural time, implementation fidelity suffers (Section 3.3.1). This recommendation rests on evidence rated as drawing primarily on correlational and mixed-methods designs for Macro-theme 1 (Section 3.2, risk of bias by synthesis), rather than on one of the five formally GRADE-rated quantitative findings; it should be read as a plausible, well-supported inference rather than a high-certainty causal claim.
2. The choice between internal and external coaching (Section 3.3.2) should take each school’s profile into account: where administrative turnover or institutional distrust is high, external coaching better protects non-evaluative boundaries; where the institution is stable, internal coaching can draw on trust that is already established. We rate this recommendation as resting on low-certainty evidence (Section 3.2): the underlying internal-versus-external comparison was appraised as low certainty in our GRADE-informed assessment, so it is offered as a context-sensitive heuristic rather than a strong directive.
3. Expectations for immediate academic outcomes should be recalibrated: given the sleeper-effect pattern identified in Macro-theme 4 (Section 3.3.4), demanding standardized literacy or mathematics gains within the same follow-up window as socio-behavioral gains may lead to prematurely abandoning programs that are, in fact, working. This recommendation follows directly from a finding rated as moderate certainty (Section 3.2), the null-to-marginal short-term academic-outcome pattern, and is therefore offered with more confidence than Recommendation 2 above.

### 4.4. Limitations

The limitations of this synthesis derive largely from the same vulnerabilities of the corpus (Section 3.2): the infeasibility of blinding, differential attrition in longitudinal designs, and the geographic concentration in the United States (Section 4.2) limit how far the findings can be generalized to other resource configurations, school cultures, or regulatory frameworks. The mid-screening broadening of eligible designs (Section 2.1) also carries a residual risk of selection bias, since reviewers were not formally blinded to study findings when the amendment was adopted; we mitigated this by applying the broadened criteria uniformly to all records rather than selectively, but we cannot rule out this risk entirely. The thematic synthesis of the qualitative and mixed-methods components is, moreover, interpretive by nature: the sleeper-effect pattern in Macro-theme 4 (Section 3.3.4) is a reasoned inference from what was observed in the corpus, not a finding proven with longitudinal designs expressly built for that temporal sequence. Future reviews should test it with longer follow-ups. A further limitation concerns the substantial methodological heterogeneity of the primary studies (coaching models, fidelity instruments, comparison conditions, and outcome measures; Section 2.7.2) and the consequent decision not to conduct a meta-analysis: in the absence of a pooled effect estimate, this review cannot provide a single quantitative answer to whether coaching is effective for SEL implementation, and the conclusions offered here describe a consistent narrative pattern across studies rather than a statistically pooled effect. Finally, the corpus includes one deliberate exception to the registered ISCED 0–2 population criterion: Longhitano (2021), a high-school (ISCED 3) lesson-study case (Section 2.2), retained for the methodological richness of its coaching model. To make this explicit rather than merely asserted, we re-examined the thematic conclusions of Section 3.3 after removing Longhitano (2021) from the corpus: all four macro-themes, including the specific claims illustrated with this study in Macro-theme 2 (Section 3.3.2), remained supported by at least one other study, and the sensitivity analysis restricted to low-risk-of-bias studies (Section 3.2) did not rely on this case in the first place. We therefore judge that its inclusion does not materially shift the thematic pattern, but readers should not interpret the synthesis as evidence that the active-ingredients framework has been validated at the secondary level.

## 5. Conclusions

This convergent segregated mixed-methods synthesis of 36 primary studies suggests that coaching and consultation are most effective when they combine an active architecture of technical components (observation, modeling, feedback, goal setting) with a non-evaluative consultative alliance, and when the school ecology allows it. The concept of adaptive fidelity proposed in this Discussion (Section 4.1) offers a framework for reconciling technical rigor with cultural responsiveness, particularly relevant when bringing these models to lower-resource, more culturally diverse contexts, including the Global South. Education policies seeking to benefit from these findings must, above all, guarantee the time and structural support that make the practice-based coaching cycle possible.

## Figures and Tables

**Table 1:** PICO eligibility framework.

PICO Element	Specifications
P—Population	In-service teachers delivering instruction at ISCED levels 0–2 (early childhood education, preschool, pre-K, kindergarten, primary education, and lower secondary education). Pre-service teachers, teacher candidates, and university-level teacher educators were excluded.
I—Intervention	Teacher coaching or consultation programs aimed at implementing a specific social-emotional learning (SEL) program or explicitly described socioemotional teaching practices, including at least two of the six explicit active components: modeling, guided practice, reflection, observation, goal setting, and performance feedback.
C—Comparison	Less specific, lower-intensity support; conditions limited solely to training; absence of structured follow-up; or business-as-usual conditions.
O—Outcomes (primary)	Fidelity of implementation, and the frequency and quality of teachers’ delivery of the SEL program.
O—Outcomes (secondary)	Students’ socioemotional, behavioral, and academic outcomes following coaching-supported implementation.

**Table 2:** Exclusion-code catalog applied during Phase 1 and Phase 2 selection.

Code	Reason for Exclusion
E1	Ineligible population (i.e., the wrong population category was studied; this code does not refer to an insufficient sample size) (pre-service teachers; teacher candidates; higher-education instructors; trainers whose own practice, rather than teachers’, is the primary target).
E2	Inadequate intervention (absence of teacher coaching/consultation with ≥2 active components; one-off training without contextualized follow-up; generic pedagogical advising without an SEL focus; consultation targeting non-teaching staff).
E3	Inadequate setting (not formal basic education or a school-based setting; non-formal or higher education).
E4	Inadequate full-text study design (secondary research; editorial, opinion, or protocol article without empirical data).
E5	Inadequate outcome (no reporting of implementation fidelity, dosage, quality, or student outcomes in SEL, behavior, or academic performance).
E6	Outside the publication window (before 1 January 2010 or after 23 June 2026).
E7	Full text inaccessible despite a reasonable retrieval effort; or duplicate undetected during deduplication.

**Table 3:** Data elements extracted from each included study.

Domain	Variables Extracted
Identification	Author(s), year, country, language, source database, identifier (DOI/OID).
Design	Study type, methodological framework, data collection and analysis techniques.
Participants	Number and characteristics of teachers, educational level, sociodemographic and geographic context.
Intervention	SEL program; coaching model; active components present (modeling, guided practice, reflection, observation, goal setting, performance feedback); duration, intensity, and format (in-person/virtual; individual/group; internal/external coach).
Comparison	Stated control or comparison condition, if any.
Outcomes	Implementation indicators (fidelity, dosage, quality); student outcomes; measurement instruments; effect magnitude where reported.
Methodological quality	Item-level MMAT 2018 appraisal by study-design category.
Context	Theoretical framework, reflexivity statement (qualitative studies), funding, and conflict-of-interest disclosures.

**Table 4:** Characteristics of the 36 included studies. M = Modeling; GP = Guided practice; R = Reflection; GS = Goal setting; O = Observation; PF = Performance feedback. Full details for each extracted variable are reported in the Supplementary Materials (Tables S1–S6) and the underlying dataset in the OSF repository (Da-ta_Extraction sheet, project M3846).

Study	Country	Design	N (Teachers)	Active Components	Coach	Key Finding
Arias de Sanchez and Li (2025)	Canada	Qual. (action res.)	18 ECEs	R, GS	External	N/A (qualitative)
Berry et al. (2016)	UK	Cluster RCT	183 classes/teachers	M, O, PF	External	No significant differences on the SDQ at 1 and 2 years
Bottiani et al. (2026)	US	Cluster RCT	64 teachers in the R-CITY …	GP, O, PF	External	Significant effects on 1/6 observational measures (physical aggression) and 1/3 self-report measures (general self-efficacy)
Cramer et al. (2021)	US	Descriptive/pilot	230	M, GP, R, GS, O, PF	External	Baseline commitment consistently predicted fidelity across time
Davies-Mercier et al. (2023)	US (Maine)	Mixed methods	28 teachers surveyed	R, GS, O	External	N/A (qualitative/descriptive evaluation; the authors caution about the small sample size)
Domitrovich et al. (2010)	US (Central Pennsylvania)	RCT (secondary)	Not reported as a single teacher-level N …	M, GP, O, PF	External	Moderate-to-strong correlations between implementation dimensions
Downer et al. (2018)	US	RCT	Not reported as a single figure …	R, O, PF	External	LOOK impacts reported on teacher strategies, self-efficacy, and child engagement (specific statistics not detailed in the summary)
Doyle et al. (2023)	US	Quasi-exp.	Not reported as a single figure …	M, R, GS, O, PF	External	Significantly lower levels of hostile attribution bias/aggression
Fox et al. (2011)	US	SCED	3	O, PF	External	Functional relation between the PD intervention (workshops+materials+guides+coaching with feedback) and implementation
Franko et al. (2025)	US (Colorado)	Qual. (case study)	Not reported as a single figure …	M, R, GS	External	N/A (qualitative case study)
Garcia et al. (2022)	US (Tennessee)	Cluster RCT	Not consolidated as a single N …	GS, O, PF	External	Small, non-significant effects on math skills (PreK) and social problem-solving (K)
Giordano et al. (2021)	US	Quasi-exp.	15	O, PF	Both (study variable)	Internal (peer) coaches more effective at supporting implementation fidelity
Hardy et al. (2025)	US	Descriptive/pilot	Not reported as a single figure …	O, PF	External	Total CQC significantly correlated with TPOT change
Harrington (2024)	US	Mixed methods	Cycle 1: 6 educators interviewed …	M, GP, R, GS, O	Internal	Only descriptive/graphical pre–post comparison reported (n = 4 teachers)
Hemmeter et al. (2015)	US	SCED	3	M, GP, R, GS, O, PF	External	Functional relation established (9 demonstrations/replications)
Hemmeter et al. (2021)	US	Cluster RCT	92	R, GS, O, PF	External	Statistically significant and notable effects on social skills and challenging behavior (focal and non-focal)
Hunter et al. (2022)	US	Mixed methods	63 teachers in 37 community centers …	GS, O, PF	Mixed (director = internal)	Teacher characteristics explained 40–45% of variance in delivery quality
Jennings et al. (2017)	US	Cluster RCT	224	R	External	Significant direct effects on adaptive emotion regulation, mindfulness, psychological distress, and time urgency
Johnson (2019)	US (Minnesota)	Descriptive/pilot	132	R, GS, O, PF	External	Significant differences in fidelity by program type, both at baseline and in change following training and coaching
Kadik et al. (2025)	US	Descriptive/pilot	202	M, GP, R, O	External	Significant pre–post improvements in classroom climate/management and in child protective factors/behavior problems (n = 138 …)
Longhitano (2021)	US	Mixed methods	5 Common Core Algebra I teachers …	M, GP, R, GS, O, PF	Internal	Only descriptive, individual-level pre/post score changes reported (e.g., one teacher’s mean DFT rating rose from 3.2 to 3.5)
Mattera et al. (2013)	US	RCT	Not consolidated as a single N …	M, GP, R, GS, O, PF	External	Moderate-to-large effect sizes on teacher practices (0.30 to 0.92, first-order)
Meyers et al. (2015)	US	Quasi-exp.	Not numerically specified	M	External	No quantitative effect size or statistical outcome reported
Morris et al. (2013)	US	RCT	Not consolidated as a single N …	M, R, O	External	Improvements in teacher classroom management in focal areas
Partee et al. (2023)	US (Virginia)	Mixed methods	Not consolidated as a single N …	GP, R, GS, O, PF	External	N/A (the article focuses on qualitative analysis of implementation tensions, not on effect sizes)
Paxton et al. (2013)	US (Mid-Atlantic)	Mixed methods	6 schools (3 high/3 low fidelity)	M, R, O, PF	External	d = 5.14 in fidelity difference between high/low schools (t(4) = 8.38, *p* = 0.001)
Rakap and Balikci (2025)	Turkey	SCED	3	GP, GS, O, PF	External	Functional relation demonstrated via visual analysis (not a traditional statistical effect size)
Robbie (2021)	US	SCED	4 novice teachers in 2 dyads	M, R, GS, O, PF	Internal	Low-to-moderate effect (TAU-U = 0.18, *p* = 0.02)
Siller et al. (2024)	US	Descriptive/pilot	25	R, O, PF	External	9/10 feasibility benchmarks met; descriptive gains reported in active engagement
Skåland et al. (2023)	Norway	Qual. (nested in RCT)	Convenience sample of teachers …	GP, R	Internal	N/A (qualitative)
Spacciapoli et al. (2022)	US	Mixed methods	301 PreK and Kindergarten teachers …	R, GS, O, PF	External	Descriptive (growth visualized through an indicator ‘tree,’ no inferential statistics)
Stoiber et al. (2022)	US	Quasi-exp.	2 public kindergarten teachers …	GP, R, GS, O, PF	External	Moderate-to-strong pre–post changes (described qualitatively)
Wang et al. (2024)	US	Quasi-exp.	Not reported as single N	O, PF	External	Baseline data presented; impact estimates not yet available in the reviewed implementation report
Wanless et al. (2013)	US (Mid-Atlantic)	Mixed methods	Study 1: 33 third-grade teachers …	M, PF	External	N/A (qualitative)
J. A. Welsh et al. (2024)	US	RCT	Not consolidated as a single N …	M, GS	Internal	Reported improvement in teacher practices vs. control
J. Welsh et al. (2025)	US	Mixed methods	Exact figure not reported	O, PF	External	Descriptive (gradual improvement in sub-component implementation across the year)

**Table 5:** Joint display of qualitative and quantitative evidence by macro-theme.

Macro-Theme	Qualitative Findings	Quantitative Findings	Integrated Interpretation
Macro-theme 1: Input ecology (systemic conditions and individual disposition)	School-leadership support and protected time are described as the primary regulators of fidelity; educator commitment to implement predicts delivery; high-need or disrupted contexts absorb teachers’ bandwidth (Wanless et al., 2013; Johnson, 2019; Cramer et al., 2021; Hunter et al., 2022; Harrington, 2024; Robbie, 2021).	Cramer et al. (2021): baseline commitment consistently predicted fidelity across time. Hunter et al. (2022): teacher characteristics explained 40–45% of variance in delivery quality; work-environment factors explained 18–41%.	Convergent—the quantitative variance-explained estimates corroborate the qualitative account that systemic and individual “input” conditions, not technical coaching design alone, are a primary driver of fidelity.
Macro-theme 2: Active architecture of coaching and the consultative alliance	Iterative observation–modeling–feedback–goal-setting cycles (Practice-Based Coaching) are consistently linked to fidelity; a non-evaluative consultative alliance is described as a necessary condition; asynchronous video formats show relational dilution (Fox et al., 2011; Hemmeter et al., 2015; Snyder et al., 2015; Hardy et al., 2025; J. A. Welsh et al., 2024; J. Welsh et al., 2025).	Hemmeter et al. (2015) and Rakap and Balikci (2025): significant fidelity gains with coaching plus performance feedback versus training only. Hardy et al. (2025): total Coaching Quality (CQC) score significantly correlated with TPOT change (82% of CQC variability attributable to the coach).	Convergent—the CQC–TPOT correlation quantitatively substantiates the qualitatively identified centrality of relational and coaching quality, not technical content alone, in producing fidelity gains.
Macro-theme 3: Scaling tensions—fidelity, cultural adaptation, and equity	Rigid, manualized fidelity is described as eroding teacher commitment in culturally heterogeneous classrooms; equity-focused supplements are framed as broadening relational reach without diluting active ingredients (Arias de Sanchez & Li, 2025; Partee et al., 2023; J. Welsh et al., 2025).	Bottiani et al. (2026): the R-CITY equity supplement was associated with a significant reduction in physical aggression (1 of 6 observational measures) and increased general teacher self-efficacy (1 of 3 self-report measures) relative to standard Second Step.	Partially convergent—the single available quantitative trial supports the qualitative claim that equity-focused adaptation can coexist with active-ingredient fidelity, but the small number of significant comparisons signals that this quantitative support is preliminary.
Macro-theme 4: Translational cascade—from teacher behavior to student impact	Proximal teacher-practice and classroom-climate gains are described as preceding and enabling distal student gains, which emerge later and inconsistently (a “sleeper effect”) (Downer et al., 2018; Siller et al., 2024; Doyle et al., 2023).	Teacher-self-report designs show academic gains (Doyle et al., 2023), while independently or standardized-assessed designs show null or marginal short-term effects (Berry et al., 2016; Hemmeter et al., 2021; Morris et al., 2013; Garcia et al., 2022).	Divergent by measurement type, not by substantive disagreement—the reporter-dependent split in the quantitative evidence is the empirical signature that motivates, rather than contradicts, the qualitative sleeper-effect interpretation.

## Data Availability

A condensed, per-study breakdown of all extracted variables is provided in Tables S1–S10 of the Supplementary Materials. The underlying data supporting the findings of this review—including the completed Phase 1 and Phase 2 screening-and-extraction workbooks (title/abstract and full-text screening decisions with exclusion codes and arbitration records; the data extraction matrix; and the item-level MMAT 2018 appraisals underlying Table 4 and Supplementary Tables S6 and S8), the original bibliographic export files, the custom Python deduplication script referenced in Section 2.4, ATLAS.ti coding files, the completed PRISMA 2020 checklist, and the full protocol version history—are openly available in the Open Science Framework repository at https://doi.org/10.17605/OSF.IO/M3846.

## Supplementary Materials

The following supporting information can be downloaded at https://www.mdpi.com/article/10.3390/bs16091513/s1: Table S1: Identification and Methodological Design; Table S2: Data Collection/Analysis Techniques, Participants, and Context; Table S3a: Intervention and Active Components of Coaching; Table S3b: Format Structure and Measurement Instruments; Table S4: Comparison Conditions, Reliability, and Results; Table S5: Theoretical Framework, Reflexivity, Funding, and Conflict of Interest; Table S6: MMAT Categorization Scores; Table S7: Summary of Qualitative Findings (GRADE-CERQual); Table S8: Item-Level MMAT 2018 Risk-of-Bias Ratings by Design Category; Table S9: Borderline Records Excluded at Full-Text Screening after Initial Reviewer Disagreement; Table S10: GRADE-Informed Certainty Assessment of Key Quantitative Findings; Table S11: Full Database Search Strategies (Scopus, Web of Science, ERIC); Table S12: Protocol Amendments Documented and Justified in the Registered Protocol Text.

## Abbreviations

The following abbreviations are used in this manuscript:

A.P.C	Alfredo Puican Carreño
C.V.C.C	Claudia Virginia Cortez-Chavez
DFT	Danielson’s Framework for Teaching
ECEs	Early Childhood Educators
ENTREQ	Enhancing Transparency in Reporting the synthesis of Qualitative research
ISCED	International Standard Classification of Education
GRADE-CERQual	Confidence in the Evidence from Reviews of Qualitative research, usando el enfoque GRADE
JBI	Joanna Briggs Institute
J.D.D.C	Juan Diego Dávila Cisneros
L.I.P.S.	Lina Iris Palacios-Serna
M.R.S.C	Mary Roxana Salazar Calderón
OSF	Open Science Framework
PD	Professional development
PRISMA	Preferred Reporting Items for Systematic Reviews and Meta-Analyses
R-CITY	Reducing Racism and Violence through Collaborative Intervention with Teachers and Youth
SDQ	Strengths and Difficulties Questionnaire
TAU-U	Nonoverlap effect size for single-case designs (Kendall’s Tau + Mann–Whitney U)
