# Supplementary material for: Teacher Coaching and Consultation with Active Components for the Implementation of Social–Emotional Learning Programs in Basic Education: A Systematic Review with Thematic Synthesis
Source: Behav Sci (Basel). 2026 Aug 28;16(9):1513. doi: 10.3390/bs16091513 (PMC13603722; doi:10.3390/bs16091513)
Supplement: Supplementary file 1 [file behavsci-16-01513-s001.zip › behavsci-4479051-supplementary.pdf]

# Supplementary Material

*Teacher Coaching and Consultation with Active Components for the Implementation of Social–Emotional Learning Programs in Basic Education: A Systematic Review with Thematic Synthesis*

This document accompanies the main manuscript and provides, for each of the 36 included studies, a complete breakdown of the variables extracted according to the form described in Table 3 of the manuscript. Table 4 in the main text presents a condensed version (author/year, country, design, N, active ingredients, key finding); Tables S1 through S6 that follow report all of the extracted variables, including the complete sociodemographic context, measurement instruments, theoretical framework, statement of reflexivity, and statements regarding funding and conflicts of interest; Table S7 reports the GRADE-CERQual assessment of the qualitative-strand review findings. The studies are listed alphabetically by first author, in the same order as Table 4 of the main manuscript, to facilitate cross-referencing. The data are drawn directly from the data extraction and methodological quality assessment spreadsheet stored in the project's OSF repository (M3846, osf.io/m3846).

## Table S1. Identification and Methodological Design

| Study | ID | Country | Language | Database | DOI / OID | Study Design | Methodological Framework | MMAT Category |
|---|---|---|---|---|---|---|---|---|
| Arias de Sanchez & Li (2025) | CORPUS-0080 | Canada | English | Scopus | 10.3390/educsci15091193 | Qualitative action research (3 cycles: plan-act-observe-reflect) | Interpretivist and positivist paradigms; action-research methodology | 1 (Qualitative) |
| Berry et al. (2016) | CORPUS-0016 | United Kingdom | English | Scopus | 10.1007/s12310-015-9160-1 | School-level cluster randomized controlled trial (RCT) | Effectiveness and micro-costing analysis (quantitative) | 2 (RCT) |
| Bottiani et al. (2026) | CORPUS-0118 | United States | English | Scopus | 10.1007/s11121-026-01907-1 | School-level cluster randomized controlled trial (RCT) | Youth-violence prevention framework with a racial-equity focus | 2 (RCT) |
| Cramer et al. (2021) | CORPUS-0091 | United States | English | Scopus | 10.1016/j.jsp.2021.08.003 | Quantitative correlational secondary analysis (two-level models, teachers nested within centers) of the Head Start CARES RCT dataset | Social-psychological theories of commitment/behavioral intention (Theory of Planned Behavior; Health Action Process Approach) | 4 (Quantitative descriptive) |
| Davies-Mercier et al. (2023) | CORPUS-0255 | United States (Maine) | English | ERIC | Not reported (ED652196, SRI Education report) | Mixed-methods evaluation (surveys + interviews + focus groups), predominantly qualitative | ECCP model of infant and early childhood mental health consultation | 5 (Mixed) / 4+1 |
| Domitrovich et al. (2010) | CORPUS-0050 | United States (Central Pennsylvania) | English | Scopus | 10.1016/j.ecresq.2010.04.001 | Randomized clinical trial (secondary analysis of implementation data from the Head Start REDI RCT, Bierman et al. 2008) | Domitrovich et al. (2008) framework: fidelity, generalization, and engagement | 4 (Quantitative descriptive) |
| Downer et al. (2018) | CORPUS-0043 | United States | English | Scopus | 10.1007/s12310-017-9237-0 | Small randomized controlled trial (RCT) | LOOK consultation model: observing child engagement in context + analyzing one's own teaching role | 2 (RCT) |
| Doyle et al. (2023) | CORPUS-0101 | United States | English | Scopus | 10.1016/j.tate.2023.104232 | Quasi-experimental (comparison of 4Rs+MTP vs. 4Rs only) | MyTeachingPartner coaching model based on CLASS | 3 (Quantitative nonrandomized) |

| | | | | | | | | |
|---|---|---|---|---|---|---|---|---|
| Fox et al. (2011) | CORPUS-0006 | United States | English | Scopus | 10.1177/0271121411404440 | Multiple-baseline single-case design, replicated across 3 teachers; with a minor end-of-study social-validity interview component | Applied behavior analysis; Teaching Pyramid Model | 5 (Mixed) / 3+1 |
| Franko et al. (2025) | CORPUS-0213 | United States (Colorado) | English | ERIC | 10.1002/imhj.70008 | Qualitative case study (development of a statewide model) | Field-expert consensus framework on essential elements of IECMHC | 1 (Qualitative) |
| Garcia et al. (2022) | CORPUS-0198 | United States (Tennessee) | English | ERIC/SREE | Not reported (technical report ED658704 / SREE series) | Two cluster randomized controlled trials (RCTs): PreK (school-level assignment) and K (teacher-level assignment) | Implementation and impact evaluation (quantitative) | 2 (RCT) |
| Giordano et al. (2021) | CORPUS-0053 | United States | English | Scopus | 10.1080/10901027.2020.1782545 | Quasi-experimental, comparison of two conditions (internal vs. external coaching) | Practice-Based Coaching (PBC); Pyramid Model PBIS framework | 3 (Quantitative nonrandomized) |
| Hardy et al. (2025) | CORPUS-0123 | United States | English | Scopus | 10.1016/j.ecresq.2025.01.003 | Correlational secondary analysis of data from a Pyramid Model RCT | Coaching Quality Framework (Hardy et al., 2016) | 4 (Quantitative descriptive) |
| Harrington (2024) | CORPUS-0181 | United States | English | ERIC | ED651823 | Qualitative action research (2 cycles: needs assessment, then PD + peer-observation intervention), with an embedded quantitative-descriptive component (pre/post self-evaluation surveys, analyzed descriptively/graphically, no inferential statistics) | Action research (Herr & Anderson, 2015); Dissertation-in-Practice model; CASEL SEL competency framework; TESOL/ESL strategy frameworks | 5 (Mixed) / 1+4 |
| Hemmeter et al. (2015) | CORPUS-0264 | United States | English | ERIC | 10.1177/0271121415594924 | Multiple-probe single-case design, replicated across teachers | Applied behavior analysis; Pyramid Model/Practice-Based Coaching framework | 3 (Quantitative nonrandomized) |
| Hemmeter et al. (2021) | CORPUS-0062 | United States | English | Scopus | 10.1016/j.ecresq.2020.08.004 | School-level cluster randomized controlled trial (RCT) | Practice-Based Coaching (Snyder et al., 2015) | 2 (RCT) |
| Hunter et al. (2022) | CORPUS-0020 | United States | English | Scopus | 10.3389/fpsyg.2022.1023505 | Mixed methods (quantitative regression + open-ended qualitative interview), correlational/predictive | Framework on teacher and work-environment factors in implementation fidelity | 5 (Mixed) / 4+1 |
| Jennings et al. (2017) | CORPUS-0277 | United States | English | ERIC | 10.1037/edu0000187 | School-level cluster randomized controlled trial (RCT) | Mindfulness-based professional development program | 2 (RCT) |
| Johnson (2019) | CORPUS-0040 | United States (Minnesota) | English | Scopus | 10.1080/03004430.2017.1331219 | Exploratory multi-site quantitative study, no control group | PBIS/Pyramid Model framework; Practice-Based Coaching (Snyder et al., 2015) | 4 (Quantitative descriptive) |
| Kadik et al. (2025) | CORPUS-0214 | United States | English | ERIC | 10.1002/imhj.22026 | Quantitative, pre-post without a control group (paired t-tests) | Three-tiered infant and early childhood mental health consultation (IECMHC) model | 3 (Quantitative nonrandomized) |

| | | | | | | | | |
|---|---|---|---|---|---|---|---|---|
| Longhitano (2021) | CORPUS-0223 | United States | English | ERIC | ED619726 | Mixed-methods (embedded) case study of a lesson-study/instructional-coaching cycle: qualitative strand (interviews, PD/planning/debrief transcripts, open-ended responses) dominant, with an embedded quantitative-descriptive strand (pre/post Likert self-ratings, n=5 teachers; explicitly underpowered student-survey component per the author) | Lesson study (Lewis et al., 2009; Lewis & Takahashi, 2013; Lewis & Perry, 2014); Danielson's Framework for Teaching (DFT); transformational learning theory for adults (Cox, 2015); Bandura's (1977) self-efficacy theory; collective teacher efficacy | 5 (Mixed) / 1+4 |
| Mattera et al. (2013) | CORPUS-0286 | United States | English | ERIC | Not reported (ED546640, MDRC/OPRE report 2013-47) | Center-level randomized controlled trial (RCT) with 3 intervention arms + control | Large-scale ('scale-up') implementation framework for evidence-based SEL programs | 2 (RCT) |
| Meyers et al. (2015) | CORPUS-0073 | United States | English | Scopus | 10.1080/10474412.2014.929956 | Case study / formative program evaluation describing a 5-stage ecological model of organizational consultation (Entry, Problem Definition, Needs Assessment, Intervention, Evaluation); reports the consultation process, not outcome results. A matched, randomly-assigned quasi-experimental Year-1 outcome evaluation is described as planned, but its results are not presented in this article. | Ecological model of organizational consultation (J. Meyers et al., 2009; A. B. Meyers et al., 2012), drawing on Bronfenbrenner (1977), Lewin (1951), Schein (1988), Vygotsky (1978) | 1 (Qualitative) |
| Morris et al. (2013) | CORPUS-0291 | United States | English | ERIC | Not reported (ED540680, MDRC report) | Center-level randomized controlled trial (RCT), replicated in two cities | Classroom-consultation model based on The Incredible Years Teaching Pyramid | 2 (RCT) |
| Partee et al. (2023) | CORPUS-0086 | United States (Virginia) | English | Scopus | 10.3389/feduc.2023.1070591 | Implementation study, mixed methods (quantitative log data + qualitative focus groups) | Domitrovich et al. (2008) implementation framework: macro-, school-, and individual-level | 5 (Mixed) / 4+1 |
| Paxton C.L.C. et al. (2013) | CORPUS-0298 | United States (Mid-Atlantic) | English | ERIC | Not reported (ED563070, SREE) | Sequential mixed methods, sub-sample (6 high/low fidelity schools) within a larger RCT | Domitrovich et al. (2008) framework: fidelity, dosage and quality | 5 (Mixed) / 4+1 |
| Rakap & Balikci (2025) | CORPUS-0023 | Turkey | English | Scopus | 10.1007/s10864-024-09551-x | Multiple-baseline single-case experimental design | Applied behavior analysis; Practice-Based Coaching (PBC) | 3 (Quantitative nonrandomized) |
| Robbie (2021) | CORPUS-0317 | United States | English | ERIC | ED620856 | Single-case experimental design: non-concurrent multiple-baseline-across-participants (2 dyads, N=4 novice teachers), with an embedded qualitative social-validity component (end-of-study interviews) | Applied behavior analysis (Cooper et al., 2007); implementation science (Fixsen et al., 2005); evidence-based classroom management (EBCM)/PBIS literature; Darling-Hammond et al. (2017) core features of effective teacher professional development | 5 (Mixed) / 3+1 |
| Siller et al. (2024) | CORPUS-0030 | United States | English | Scopus | 10.1177/13623613231179289 | Uncontrolled feasibility trial (quantitative, single group) | Appreciative Inquiry framework applied to classroom-video reflection | 3 (Quantitative nonrandomized) |

| | | | | | | | | |
|---|---|---|---|---|---|---|---|---|
| Skåland et al. (2023) | CORPUS-0025 | Norway | English | Scopus | 10.1186/s40359-023-01088-4 | Qualitative, semi-structured pre/post interviews nested within a larger RCT | Theory of Planned Behavior | 1 (Qualitative) |
| Spacciapoli et al. (2022) | CORPUS-0019 | United States | English | Scopus | 10.3389/feduc.2022.1020204 | Feasibility pilot study, mixed methods (quantitative + qualitative) | Implementation fidelity framework (Dusenbury et al., 2003); Vygotskian theory | 4 (Quantitative descriptive) |
| Stoiber et al. (2022) | CORPUS-0026 | United States | English | Scopus | 10.1080/10474412.2021.1984930 | Quasi-experimental, no comparison group (exploratory, pre-post) | Tier I PBIS consultation model; Coaching Quality Framework | 3 (Quantitative nonrandomized) |
| Wang et al. (2024) | CORPUS-0197 | United States | English | ERIC | 10.7249/RRA3269-1 | Quasi-experimental implementation evaluation (mixed methods) | PEDALS model: SEL curriculum + coaching support + child assessment + data use + sustainability planning | 5 (Mixed) / 3+1 |
| Wanless et al. (2013) | CORPUS-0076 | United States (Mid-Atlantic) | English | Scopus | 10.1007/s11121-012-0294-1 | Sequential mixed methods (2 studies: focus groups + quantitative/qualitative validation) | Ecological setting-level framework for implementation fidelity | 1 (Qualitative) |
| Welsh et al. (2024) | CORPUS-0119 | United States | English | Scopus | 10.1016/j.sel.2024.100036 | Randomized controlled trial (RCT) at the center level | Adapted professional-development framework (online + director coaching) | 2 (RCT) |
| Welsh et al. (2025) | CORPUS-0183 | United States | English | Scopus | 10.1007/s10643-024-01648-2 | Implementation pilot study, mixed methods (quantitative fidelity + qualitative interviews) | Framework for fidelity and adaptation of evidence-based programs in new contexts | 5 (Mixed) / 4+1 |

*Table S1. Bibliographic information, study design, methodological framework, and assigned MMAT category (Hong et al., 2018).*

## Table S2. Data collection/analysis techniques, participants, and context

| Study | Data Collection Technique | Data Analysis Technique | N Teachers | Educational Level | Sociodemographic Context |
|---|---|---|---|---|---|
| Arias de Sanchez & Li (2025) | Separate focus groups for ECEs and coaches | Iterative thematic analysis (open coding, consensus between 2 researchers) | 18 ECEs (early childhood educators) + 10 coaches | Early childhood education (ISCED 0) | A Canadian province; rural and urban areas, English- and French-speaking communities |
| Berry et al. (2016) | SDQ; PTRS; T-POT; fidelity questionnaire completed by PATHS coaches in consultation with teachers | Intention-to-treat (ITT) analysis per CONSORT guidance for cluster trials; micro-costing analysis | 183 classes/teachers (101 teachers mentioned in the cost analysis) | Elementary, ages 4-6 (ISCED 1) | 56 primary schools in a large UK city; 5,074 children at baseline |
| Bottiani et al. (2026) | Observation of student behavior; teacher self-report survey; coaching contact logs | Sensitivity analysis excluding the most COVID-19-affected cohort; condition comparison | 64 teachers in the R-CITY condition (55 with at least 15 min of coach contact) | Elementary and lower secondary (ISCED 1-2) | 27 elementary and lower-secondary schools, randomly assigned |
| Cramer et al. (2021) | Teacher surveys; coach reports; classroom observation by trained observers | Two-level models (teacher within center), fixed effects by Head Start agency | 230 (lead teachers in 230 Head Start classrooms: 76 Tools of the Mind, 77 PATHS, 77 Incredible Years) | Head Start/Preschool (ISCED 0) | 104 Head Start centers, 17 agencies (the same study as Mattera et al. 2013, CORPUS-0286) |
| Davies-Mercier et al. (2023) | Surveys; semi-structured interviews; focus groups with teachers and parents | Descriptive analysis of surveys; thematic analysis of interviews/focus groups | 28 teachers surveyed (out of an unspecified total served across 8 pilot counties) | Early childhood education (ISCED 0), including Head Start, licensed centers, family child care and other settings | 8 Maine counties (5 initial pilot sites + 3 additional, funded by ARPA/CCDF funds) |
| Domitrovich et al. (2010) | Monthly REDI Trainer ratings (TIR); direct child assessment | Correlations between implementation dimensions and child outcomes | Not reported as a single teacher count; 22 intervention classrooms (of 44 total, 3 Head Start programs) | Head Start/Preschool (ISCED 0) | 3 Head Start programs in Central Pennsylvania; 192 four-year-old children (2 cohorts) |
| Downer et al. (2018) | Teacher-recorded classroom video every 2 weeks; child-engagement measures; teacher self-report | Group comparison (LOOK vs. control) on teacher strategies and child engagement | Not reported as a single figure; 45 Head Start classrooms, 143 children identified with disruptive behavior | Preschool/Head Start (ISCED 0) | An urban sample of preschool teachers and children at risk for challenging behavior |
| Doyle et al. (2023) | Teacher-recorded classroom video every 2 weeks; written coach feedback; teacher questionnaires; attendance | Comparison of classroom-level effects between conditions | Not reported as a single figure; 91 classrooms in 15 urban elementary schools | Elementary (ISCED 1) | 91 classrooms in 15 urban public elementary schools |
| Fox et al. (2011) | TPOT observation (2 hours + 15-20 min interview); social-validity questionnaire and interview | Visual analysis of single-case data (Horner & Baer, 1978) | 3 (early childhood special education, ECSE, teachers) | Inclusive public-school preschool (ISCED 0) | 3 inclusive classrooms across 3 elementary school campuses within the same district |
| Franko et al. (2025) | Statewide interviews with key stakeholders | Qualitative case analysis | Not reported as a single figure; statewide case study with interviews across multiple stakeholders | Early childhood education (ISCED 0) | State of Colorado, United States; IECMHC model implemented since 2006 (Boulder/Denver pilots) |

| | | | | | |
|---|---|---|---|---|---|
| Garcia et al. (2022) | Classroom observation; coaching logs; child outcome assessments | Hierarchical linear models (HLM); intent-to-treat impact analysis | Not consolidated as a single figure; 4 cohorts of PreK and K teachers in MCPS and MNPS (2018-2022) | Preschool and Kindergarten (ISCED 0) | Two Tennessee districts (one rural, one urban); diverse student population, ~1/4 multilingual learners |
| Giordano et al. (2021) | Pre/post fidelity assessment; pre/post individual child social-skills assessment | Comparison of fidelity and social-skills change between conditions | 15 (8 with internal peer coaching, 7 with external coaching) | Preschool/Head Start (ISCED 0) | Head Start classrooms; 125 participating students |
| Hardy et al. (2025) | Coding of recorded coaching sessions (CQC); classroom observation (TPOT) | Correlations; analysis of variability attributable to coach vs. teacher | Not reported as a single figure; each coach worked with 1-7 teachers (mean=3.81) over 2 years | Preschool (ISCED 0) | Sample from a Pyramid Model RCT (region not specified) |
| Harrington (2024) | Semi-structured educator interviews; MLL student focus group (facilitated by school social worker); classroom walkthrough observations; pre/post self-evaluation surveys; session exit tickets; peer-observation rubric | Interviews/focus groups transcribed and coded for emergent themes (coding scheme not fully detailed); survey/exit-ticket data displayed descriptively via bar graphs and matrices/graphics (Miles, Huberman & Saldaña, 2019) | Cycle 1: 6 educators interviewed (4 classroom teachers + 2 special educators); Cycle 2 (PD/coaching intervention): 4 third-grade classroom teachers (the intact PLC team) + 3 administrators (principal + 2 assistant principals) as non-recipient stakeholders | Elementary, Grade 3 (ISCED 1); urban public K-5 school | West View Elementary (pseudonym), urban K-5 school, ~675 students (100% free-lunch eligible), 370 identified multilingual learners (largest MLL population in the district); district under state takeover (2019) and DOJ Equal Education Opportunity Act settlement (2018) requiring district-wide ESL certification and MLL-focused PD |
| Hemmeter et al. (2015) | Team-designed observation checklists; TPOT; CCBOT; audio recording for fidelity | Visual analysis of single-case data | 3 | Preschool (ISCED 0) | Urban school district, 3 elementary schools; 87.5-93.8% free/reduced-price lunch; ~50% children with disabilities |
| Hemmeter et al. (2021) | Classroom observation (TPOT); teacher rating scales; direct child assessment | Two-level modeling in Mplus (child within teacher, teacher within school) | 92 | Preschool (ISCED 0) | 76 schools (61 with 1 teacher, 14 with 2, 1 with 3); 997 children, 250 at-risk focal children |
| Hunter et al. (2022) | Classroom observation; surveys; open-ended qualitative interview on the work environment | Multiple regression (teacher characteristics vs. work-environment factors) | 63 teachers in 37 community-based childcare centers | Preschool in childcare centers (ISCED 0) | Community-based childcare centers in the United States, variable resources and funding |
| Jennings et al. (2017) | Teacher self-report (pre/post); classroom observation (CLASS) | Multilevel models (teachers nested within schools) | 224 | Elementary, K-5 (ISCED 1) | 36 urban elementary schools; ethnically diverse sample (66.6% non-White) |
| Johnson (2019) | Structured classroom observation (TPOT) by master cadre members | Comparison of fidelity levels by program type and region | 132 (of 151 initially enrolled; observational data reported for 132) | Preschool, ages 3-5 (ISCED 0) | 32 early childhood programs across 11 Minnesota regions; 7 program types (Head Start, ECSE, ECFE, childcare, etc.) |
| Kadik et al. (2025) | Classroom observation (TPOT-SF); teacher questionnaire (C-SDQ); child assessment (DECA) | Paired pre-post t-tests; moderation analysis by child demographic attributes | 202 (one per each of the 202 classrooms that received classroom consultation) | Early childhood education, ages 2-5 (ISCED 0) | 69 subsidized early-care sites across New York City's five boroughs; 2,832 children (39.3% Hispanic, 36.2% African American) |
| Longhitano (2021) | Pre/post teacher and student Likert + open-ended surveys (Google Forms); classroom observation forms; video-recorded/transcribed semi-structured interviews, PD sessions, planning meetings, and group debrief meeting; teacher open-ended responses in shared Google Docs | Open-ended/interview/transcript data coded for emergent themes against DFT domains; quantitative survey data compared descriptively pre- to post-study (e.g., individual mean DFT self-ratings); qualitative and quantitative findings explicitly cross-compared for corroboration or contradiction | 5 Common Core Algebra I teachers (of 7 eligible) participated in the full lesson-study cycle; 1 of the 5 did not complete the peer-observation component | Upper secondary / High school (ISCED 3; included as a single deliberate extension case beyond the review's core ISCED 0–2 population — see manuscript Sections 2.2 and 4.4 for the explicit justification); Common Core Algebra I and an associated 'algebra lab' support course | Suburban NYC-area high school, 1,600 students (58% Hispanic, 23% White, 11% Black, 5.5% Asian); 53% free/reduced-lunch eligible; de-tracking reform context to address prior racial disparity in double- vs. single-period Algebra I placement; study conducted during COVID-19 hybrid instruction |

| | | | | | |
|---|---|---|---|---|---|
| Mattera et al. (2013) | Coaching logs (weekly/monthly); classroom observation by trainers | Pooled impact analysis controlling for randomization block | Not consolidated as a single figure; 307 classrooms (lead + assistant) in 104 Head Start centers, 17 agencies across 10 states | Head Start/Preschool (ISCED 0) | 104 Head Start centers in 10 U.S. states, nationally representative of Head Start setting diversity |
| Meyers et al. (2015) | Individual stakeholder interviews (community leaders, administrators, teachers, etc.); group interview with school psychologists/social workers; online administrator survey; teacher implementation-integrity checklists; teacher survey on implementation roadblocks | Narrative/descriptive synthesis of interview and survey responses; no formal qualitative coding scheme, software, or inter-rater procedure reported | Not numerically specified; general education teachers deliver the curriculum across a phased rollout of 27 school attendance centers (~7,200 students county-wide) | Basic Education (K-12) | 27 school attendance centers, ~7,200 students, rural Midwestern county (central Illinois); predominantly White (92.8%); median household income and HS-graduation rate below state average; elevated rates of adolescent pregnancy, domestic violence, child maltreatment (Voices for Illinois Children, 2007) |
| Morris et al. (2013) | Classroom observation; teacher and child ratings | Impact analysis comparing FOL vs. usual preschool | Not consolidated as a single figure; 91 classrooms in 71 preschool centers (Newark, NJ and Chicago, IL) | Preschool (ISCED 0) | Newark (better-resourced system) and Chicago (more limited institutional resources) |
| Partee et al. (2023) | Consultation logs; participant surveys; focus groups; observation (TPOT-S, inCLASS) | Descriptive implementation statistics; thematic analysis of focus groups | Not consolidated as a single figure; teachers in 45 served preschool classrooms (out of 89 referred) | Preschool (ISCED 0) | 45 classrooms across 30 programs in a large Virginia region; pilot year 2021-2022 |
| Paxton C.L.C. et al. (2013) | Structured observation (CPOM); teacher surveys (CPTS, CPFS); qualitative coach post-visit summaries | Independent-samples t-test; thematic coding of coaching summaries | Fifth-grade teams in 6 schools (3 high-fidelity / 3 low-fidelity), a sub-sample of a larger RCT (RCES) | Elementary, 5th grade (ISCED 1) | A Mid-Atlantic state, United States |
| Rakap & Balikci (2025) | Structured direct observation (checklist); session recording | Visual analysis of single-case data | 3 | Preschool (ISCED 0) | Preschool classrooms in Turkey; 3 teachers and 3 children with challenging behavior |
| Robbie (2021) | Weekly/bi-weekly 15-minute video-recorded classroom self-observations, tallied by the peer coach for general/specific praise and corrective statement counts; cumulative graphed performance-feedback data; end-of-study social-validity interviews | Visual analysis of single-case graphed data; Tau-U effect-size estimation (TAU-U = 0.18, p = .02); percentage of non-overlapping data (PND); qualitative thematic analysis of end-of-study social-validity interviews | 4 novice teachers in 2 dyads (Dyad 1: Rosanna [2nd-year, grade 4] & Reilly [4th-year, K-5 special education]; Dyad 2: Garrett [4th-year, grade 3] & Ginny [3rd-year, K-5 special education]) | Elementary (K-5/preK-6, ISCED 0–1); 4 different U.S. schools (suburban and rural, western and northeastern U.S.), Title I status for 3 of 4 sites | 4 geographically dispersed U.S. elementary schools (western and northeastern U.S.), student populations ranging 111–1,047; free/reduced-lunch rates 19–73%; conducted during the COVID-19 pandemic (recruitment July–October 2020), which severely constrained participant recruitment (target 6–10 participants in 3–5 dyads; only 4 obtained despite outreach to >5,000 contacts) |
| Siller et al. (2024) | Surveys; video-coded classroom observation; coding of fidelity for recorded coaching sessions | Descriptive statistics; assessment against a-priori feasibility benchmarks | 25 (one teacher per each of 25 preschool classrooms) | Preschool (ISCED 0) | 25 preschool classrooms (private, Head Start, public Pre-K), maximum-variation sample |
| Skåland et al. (2023) | Individual semi-structured telephone interviews | Thematic analysis | Convenience sample of teachers and kindergarten leaders (exact number not consolidated; sub-sample of 22 FUS kindergartens participating in the larger RCT) | Kindergarten/early childhood education (ISCED 0) | 22 FUS kindergartens in Norway (January-June 2020) |

| | | | | | |
|---|---|---|---|---|---|
| Spacciapoli et al. (2022) | Teacher-recorded video; indicators marked by the teacher and the curricular coach | Descriptive statistics of fidelity and longitudinal growth indicators | 301 PreK and Kindergarten teachers | Preschool and Kindergarten (ISCED 0) | 65 sites/programs across several hundred classrooms in the United States (Tools of the Mind) |
| Stoiber et al. (2022) | Structured observation (CPBO, PSIM); Goal Attainment Scaling; teacher interview/self-assessment | Pre-post comparison; inter-observer consensus ratings | 2 public kindergarten teachers + Head Start classroom teachers (15 EC classrooms total; consultation with 15 coaches) | Early childhood education/kindergarten/Head Start (ISCED 0) | 15 urban EC classrooms, predominantly African American children (99% in 14 classrooms) |
| Wang et al. (2024) | Classroom observation (2 rounds); surveys; focus groups/interviews | Descriptive statistics of fidelity/quality; propensity-score weighting (impact, ongoing) | 39 teachers, 27 teaching assistants, 16 site directors | Pre-Kindergarten (ISCED 0) | PreK classrooms in southeastern Michigan (Detroit) and western/central New York (Buffalo/Syracuse) |
| Wanless et al. (2013) | Focus groups with teachers; interviews with coaches and principals | Thematic coding; content analysis (Miles & Huberman) | Study 1: 33 third-grade teachers; Study 2: 50 fourth-grade teachers | Elementary (ISCED 1) | A large Mid-Atlantic district, United States; ~1/4 free/reduced-price lunch, ~40% White, ~25% Hispanic |
| Welsh et al. (2024) | Classroom observation; instructional quality assessment | Pre-post comparison, intervention vs. control group | Not consolidated as a single figure in the reviewed excerpt; multiple childcare centers across 10 counties | Preschool in childcare centers (ISCED 0) | 10 counties characterized by concentrated poverty (state anonymized for review) |
| Welsh et al. (2025) | Classroom observation (modified CLASS); coaching logs; post-implementation semi-structured interviews | Descriptive fidelity statistics; qualitative analysis of adaptations | Exact figure not reported; teachers in Head Start preschools on and near a Native American reservation | Preschool/Head Start (ISCED 0) | Head Start preschools on and near a Native American reservation, Indigenous and non-Indigenous students and teachers |

*Table S2. Data collection and analysis techniques, participant characteristics, and sociodemographic/geographic context.*

## Table S3a. Intervention and Active Components of Coaching

| Study | SEL Program | Coaching Model | M | PG | R | EM | O | RD | Duration | Intensity | Modality | Coach |
|---|---|---|---|---|---|---|---|---|---|---|---|---|
| Arias de Sanchez & Li (2025) | Pyramid Model | Practice-Based Coaching (Snyder et al., 2015), cited as the program's framework but without a detailed procedural description in this article | N | N | Y | Y | N | N | 2023-2025 (3 action-research cycles) | Each coach assigned to 1-5 ECEs; 3 waves of focus groups after each cycle | In person | External (PM coaches assigned by the partner organization) |
| Berry et al. (2016) | PATHS (Promoting Alternative Thinking Strategies) | PATHS coaches: on-site implementation support, fidelity data collected in consultation with teachers (mechanics not described in detail; the article focuses on cost-effectiveness) | Y | N | N | N | Y | Y | 2 academic years | 1.5 days of training workshops; ongoing implementation support from PATHS coaches (frequency not quantified) | In person | External (PATHS coaches, project team) |

| | | | | | | | | | | | | |
|---|---|---|---|---|---|---|---|---|---|---|---|---|
| Bottiani et al. (2026) | Second Step + R-CITY | R-CITY: one-on-one equity-focused coaching, a four-step protocol (shared with the SEL-only condition, with greater dosage and equity-content integration) | N | Y | N | N | Y | Y | One school year (sample affected by a COVID-19-impacted cohort) | Average of 6.11 coaching contacts per teacher; ~186 min (3.1h) of direct interaction per case | In person, one on one | External (R-CITY project coaches) |
| Cramer et al. (2021) | Head Start CARES: Tools of the Mind, Preschool PATHS, Incredible Years | Head Start CARES coaching (same as CORPUS-0286): observation + reflection + modeling + problem-solving + goal-setting and weekly plan | Y | Y | Y | Y | Y | Y | One academic year (2 cohorts 2009-2011) | Same as the primary study (CORPUS-0286): ~90 min/week of coaching with 52 coaches | In person, in the classroom | External (the same 52 coaches from the Head Start CARES study) |
| Davies-Mercier et al. (2023) | ECCP (Early Childhood Consultation Partnership) | ECCP: individualized consultation with classroom observation and action plans | N | N | Y (reflective supervision mentioned for consultants, extended to the consultative relationship with teachers) | Y | Y | N | Pilot period January 2021 - March 2023 | Individualized consultation of variable intensity and duration by case | In person and virtual | External (6 ECCP consultants employed by contracted mental-health agencies) |
| Domitrovich et al. (2010) | Head Start REDI (Research-based, Developmentally Informed) | REDI: weekly mentoring by REDI Trainers - modeling, coaching, and ongoing feedback; ~3h/week observing, demonstrating, or co-teaching | Y | Y | N | N | Y | Y | One intervention year | 3-day training + 1-day booster; weekly mentoring: ~3h/week observing/demonstrating/co-teaching + 1h/week meeting | In person | External (REDI Trainers, experienced teachers supervised by senior project trainers) |
| Downer et al. (2018) | LOOK (Learning to Objectively Observe Kids) | LOOK: online modules + guided video review deepening self-reflection and self-analysis of teaching practice, with video-based feedback | N | N | Y | N | Y | Y | One school year | Teachers film themselves every 2 weeks throughout the year implementing evidence-based strategies | Online/web-mediated (modules + web-mediated consultant contacts) | External (project consultants) |
| Doyle et al. (2023) | 4Rs (Reading, Writing, Respect & Resolution) + MTP (MyTeachingPartner) | 4Rs+MTP: an 8-step cycle - fortnightly video, CLASS-aligned segments, written feedback and questions, 30-45-minute meeting, written summary and action plan | Y | N | Y | Y | Y | Y | One school year | 8 structured one-on-one coaching cycles, every 2 weeks | Online/video-based + 30-45-minute in-person/virtual meeting per cycle | External (MTP-model coaches) |

| | | | | | | | | | | | | |
|---|---|---|---|---|---|---|---|---|---|---|---|---|
| Fox et al. (2011) | Teaching Pyramid Model | Coaching with performance feedback (Teaching Pyramid Model PD) | N | N | N | N | Y | Y | Variable per teacher (multiple baseline until criterion) | Training (workshops, materials, implementati on guides) + coaching with performance feedback until criterion | In person, individual | External (research-team coaches) |
| Franko et al. (2025) | Colorado statewide IECMHC model (HB20-1053) | Colorado IECMHC: (1) collaborative development of data-driven goals; (2) relevant coaching, training, and modeling; (3) consultative stance | Y | N | Y (the consultative stance fosters self-reflection) | Y | N | N | Model developme nt from 2020 (legislation) onward | Not quantified (a case study of policy/model development) | In person (consultat ion) and statewide policy-developm ent processes | External (early childhood mental health consultants) |
| Garcia et al. (2022) | Pyramid Model | Practice-Based Coaching (PBC), delivered by district-hired coaches (not model developers) | N | N | N | Y | Y | Y | 2 years per cohort (1 training year + 1 implementa tion year) | Weekly coaching during the training year; monthly during the implementati on year (8 sessions) | In-person, individual | External (district-hired coaches) |
| Giordano et al. (2021) | Pyramid Model | Practice-Based Coaching, comparing an internal (peer) coach vs. an external (hired) coach | N | N | N | N | Y | Y | 8 weeks | 8 weeks of coaching following Practice-Based Coaching and social-emotional-framework training | In person | Both (study variable): internal (peer) vs. external (hired) |
| Hardy et al. (2025) | Pyramid Model | Practice-Based Coaching, assessed via the CQC (Foundational, Supportive, Change-Oriented subscales) | N | N | N | N | Y | Y | 2-year intervention | 168 coded coaching sessions (156 analyzed, sessions 3-15 of a 16-session cycle) | In person, individual | External (coaches from the Pyramid Model RCT) |

| | | | | | | | | | | | | |
|---|---|---|---|---|---|---|---|---|---|---|---|---|
| Harrington (2024) | Researcher-designed SEL/ESL professional-development series (no named commercial curriculum); delivered in partnership with Center for Resilience (community mental-health/mindfulness organization) | PLC-embedded professional-development and peer-observation model, with the researcher acting as facilitator/coach, incorporating CASEL SEL standards and TESOL/ESL protocols (ELLevations platform) | Y ('Teachers were led through the lesson by the researcher modeling effective strategies for MLLs'; ESL protocols explicitly modeled before teachers used them) | Y (teachers rehearsed strategies during PLC sessions by 'playing the role of the student, engaging in hands-on learning' before classroom application) | Y (session exit tickets; PLC discussion; peer-observation recording sheet explicitly described as 'reflexive for educators observing the lesson and teaching the lesson'; Appendix C critical reflection) | Y ('Teachers chose one [CASEL] skill to focus on in their classroom and implement for that week') | Y (5 peer classroom observations, 30–45 min each, using a co-designed walkthrough rubric; explicitly non-evaluative) | N: arbitrated: observations were explicitly designed as non-evaluative, reciprocal, learning-oriented peer exchanges rather than performance feedback tied to an individual goal/standard from a coach; the 'what could be improved' notes were self-directed reflection prompts, not delivered feedback from an observer to the observed | Cycle 1 (needs assessment/interviews/observations) plus Cycle 2 PD series implemented over approximately 4 months (August–December) | PLC-based professional-development series (session count not exhaustively enumerated in the reviewed text; see Appendix A Table A1) plus 5 peer classroom observations (30–45 min each) in the final month | In-person (PLC sessions, modeling, peer classroom observations) | Internal (the action researcher is an insider third-grade peer teacher facilitating the PLC), with external content input from a community partner organization (Center for Resilience) |
| Hemmeter et al. (2015) | Pyramid Model | Coaching with performance feedback (Pyramid Model PD) | Y | Y | Y | Y | Y | Y | Variable per teacher (staggered across 3 sets of practices until criterion, ~1 school year) | Observation 1x/week (data) + 1x/week (non-data coaching); feedback 3x/week (70% in person, 30% email) | Hybrid (in person + email) | External (research-team coaches) |
| Hemmeter et al. (2021) | Pyramid Model | Practice-Based Coaching (PBC): focused observation + debriefing with reflection and feedback + action plan/goals + email follow-up | N | N | Y | Y | Y | Y | One school year (workshops + weekly coaching, excluding breaks) | 16.95 coaching sessions on average (SD=0.22); observations ~91 min; debriefing ~33 min | In person + email follow-up | External (project research-team coaches) |
| Hunter et al. (2022) | REDI (adapted for childcare centers) | Adapted REDI: center directors as local coaches, supervised by regional REDI consultants | N | N | N | Y | Y | Y | One school year (October to April) | REDI consultant visits 2x/month (first 2 months), then monthly; regular director-teacher meetings | In person; hybrid with online modules for initial training | Mixed (director = internal coach; REDI consultant = monthly external technical support) |

| | | | | | | | | | | | | |
|---|---|---|---|---|---|---|---|---|---|---|---|---|
| Jennings et al. (2017) | CARE for Teachers (Cultivating Awareness and Resilience in Education) | CARE for Teachers: individual telephone coaching calls following group mindfulness training | N | N | Y | N | N | N | 5 days of training between November and February + coaching calls during intersession breaks | 30 hours of in-person training + 3 individual telephone coaching calls (~26 min average each) | In person (training) + telephone (coaching) | External (program facilitators/fidelity coders) |
| Johnson (2019) | Pyramid Model (PBIS framework) | Practice-Based Coaching: opening, observation summary/reflection, supportive and constructive feedback, targeted support, action plan and resources, closing | N | N | Y | Y | Y | Y | 2-year cohorts (state-level professional development system) | 6-9 days of training + monthly (minimum) coaching with TPOT observation | In person + monthly support teleconferences | External (state department of education master cadre members) |
| Kadik et al. (2025) | IECMHC - NYC Early Childhood Mental Health Network | IECMHC: classroom consultation - reflective discussions, coaching on behavior-management techniques, modeling of positive relationships | Y | Y | Y | N | Y | N | Average consultation period of ~7.5 months | Consultant on site 1 day/week per site, focused on 3 classrooms per site | In person, on site | External (35 master's-level mental health consultants, employed by community clinics) |
| Longhitano (2021) | 'SSATSEL' algebra-lab curriculum model integrating Skills Support, Authentic Tasks, and Social-Emotional Learning (not a named commercial SEL curriculum; SEL is one of 3 pillars co-designed via lesson study) | Lesson study (Japanese-origin PD model) facilitated by a mathematics instructional coach (Knight, 2007 model), structured through the lens of Danielson's Framework for Teaching | Y (instructional coach models effective practice during PD/planning; coaching received by at least one teacher explicitly tied to specific DFT domains, e.g., Julie 'received coaching from [coach] specifically to improve' in named domains) | Y: arbitrated: collaborative lesson/task design and refinement in planning meetings, with the coach present and iterating live with teachers, constitutes guided practice in the design/planning sense, though it is weaker evidence than in-classroom real-time coaching | Y (structured post-observation group debrief meetings explicitly transcribed and analyzed, e.g., 'Discussion. Andrew and Leah both noted...'; self-rating survey reflection pre/post) | Y (individual coaching explicitly targeted specific DFT domains per teacher, e.g., Julie's coaching goals in Domain 3 sub-components) | Y (classroom intervisitation/peer observation of lessons, using structured observation forms, for 4 of 5 teachers) | Y (individualized, domain-specific coaching feedback explicitly described, e.g., coaching tied to Domains 3b/3c/3e for a named teacher, with a documented before/after DFT rating change) | One school year (pre-study baseline through post-study/exit interviews); instructional coach worked with administrators the prior year and over the summer to design the model | Pre-study interview + survey, professional-development session(s) with the instructional coach, lesson-study planning meetings, 1 peer observation, post-study survey, exit interview per teacher | In-person/hybrid (COVID-19 hybrid instructional model); planning, PD, and debrief meetings conducted via Google Meet (video-recorded and transcribed) | Internal (the mathematics instructional coach, who is also the researcher, is embedded within the same high school) |

| | | | | | | | | | | | | |
|---|---|---|---|---|---|---|---|---|---|---|---|---|
| Mattera et al. (2013) | Head Start CARES: The Incredible Years, Preschool PATHS, Tools of the Mind - Play (3 'enhancements') | Head Start CARES coaching: classroom observation + joint reflection + technique modeling + problem identification/resolution + goal-setting and weekly plan | Y | Y | Y | Y | Y | Y | One academic year (2009-2011, 2 cohorts) | 90 min/week of coaching (60 min classroom observation + 30 min meeting); 52 coaches, ~4 classrooms each on average | In person, in the classroom | External (52 coaches hired with study funds, supervised by trainers) |
| Meyers et al. (2015) | Positive Action (Flay, Allred, & Ordway, 2001; Snyder et al., 2010) | Ecological organizational consultation (systems-level); not an individualized teacher-coaching model in the classroom-embedded sense | Y: with caveat: modeling emerges only as a late-stage (Year 3) adaptation via peer 'SEL champions', not as an original designed component | N (no description of teachers rehearsing the practice with real-time coach support) | N (reflection is built into the consultants' recursive process model, not into a structured teacher-facing coaching cycle) | N | N (no classroom observation of teacher SEL delivery by coaches/consultants is described) | N | 16 months of planning and 3 years of implementation | Tier I universal SEL instruction; originally >70 lessons/year, revised to core 24 lessons/year (~weekly) after teacher feedback in Year 2; teachers could infuse additional lessons ad hoc | In-person | External initially (university-based Project Evaluators/Director; county Special Services Unit consultants), transitioning toward internal peer support (teacher 'SEL champions') by Year 3 |
| Morris et al. (2013) | Foundations of Learning (FOL), based on The Incredible Years Teaching Pyramid | FOL: master's-level Classroom Consultant (CCC), weekly in-classroom support - models techniques and acts as a 'sounding board' (joint reflection) for teachers | Y | N | Y | N | Y | N | One school year (PreK year) | In-classroom support 1 day/week by a master's-level Classroom Consultant (CCC), plus training workshops | In person, in the classroom | External (master's-level, clinically trained CCC) |
| Partee et al. (2023) | Virginia ECMHC pilot (Virginia Department of Education) | Virginia ECMHC: kickoff session with self-assessment -> action-plan creation -> plan implementation (video-recorded when possible) -> consultant observation and feedback | N | Y | Y (initial self-assessment to identify improvement topics) | Y | Y | Y | One pilot year (2021-2022) | Low-intensity tier: kickoff session + 4 consultation cycles; high-intensity tier: kickoff session + 7 cycles (each cycle = session + observation) | In person and virtual | External (2 full-time + 1 part-time consultants, hired and trained by a university research center) |
| Paxton C.L.C. et al. (2013) | Responsive Classroom (RC) | Responsive Classroom (NEFC): RC1/RC2 workshops + observation visits + individual consultation | Y | N | Y | N | Y | Y | First year of RC implementation | 3 on-site training/coaching visits per year (RC1/RC2 workshops + observation + individual consultation) | In person, individual and group | External (NEFC-certified coaches) |

| | | | | | | | | | | | | | |
|---|---|---|---|---|---|---|---|---|---|---|---|---|---|
| Rakap & Balikci (2025) | Pyramid Model | Practice-Based Coaching (PBC) cycle: observation -> data-based feedback -> action plan | N | Y | N | Y | Y | Y | Variable per teacher (multiple baseline until criterion) | Weekly coaching until reaching 80% correct implementation across 3 consecutive sessions | In-person, individual (1 coach : 1 teacher) | External (research-team coach) |
| Robbie (2021) | Evidence-based classroom management (EBCM) peer-coaching intervention package targeting positive-to-negative teacher/student interaction ratios (not a named commercial SEL curriculum; grounded in PBIS/EBCM literature) | Reciprocal peer-coaching multi-component intervention package (video-based self-observation + peer-delivered performance feedback + gradual fading of support), explicitly incorporating 6 of the 7 core features of effective teacher PD (Darling-Hammond et al., 2017) | Y (explicit instruction included models of effective practice via readings/videos; peers also modeled target practices for one another through the shared videos) | N: arbitrated: practice occurred independently/asynchronously in the teacher's own classroom without the coach present during the attempt, which does not meet the codebook's 'real-time support during the attempt itself' criterion | Y (explicit 'offers feedback and reflection' design feature; end-of-study interviews further probed participant reflection on the experience) | Y: arbitrated: habit-based action-planning tools were an explicit part of the intervention package (Appendix F), and dyads were explicitly encouraged toward action planning; this meets a reasonable reading of collaborative goal-setting even though phase criteria were standardized | Y (weekly/bi-weekly video-based peer observation of actual classroom teaching, distinct from self-report) | Y (explicit, data-based performance feedback: tallied praise/corrective-statement counts and calculated interaction ratio delivered to each participant on a cumulative graph) | 16–20 weeks of active intervention per participant (Dyad 1: 10 weeks; Dyad 2: 11 weeks), preceded by a baseline phase (8–14 data points, randomly assigned length) and followed by a ~2-month faded-support maintenance phase | Weekly 15-minute video-recorded self-observations reviewed by a peer coach (tallying general/specific praise and corrective statements; ratio calculated and graphed), shifting to bi-weekly after 10–12 weeks; one 1-hour virtual training session at intervention onset | Remote/virtual (COVID-19 context): video-recorded classroom self-observation, virtual training via Zoom, peer feedback via spreadsheet/email | Internal/peer, a reciprocal peer-coaching model with no external expert coach, explicitly designed for feasibility without reliance on outside experts |
| Siller et al. (2024) | SEE-KS-EC (Social Emotional Engagement-Knowledge & Skills-Early Childhood) | SEE-KS-EC coaching: classroom-video review using an Appreciative Inquiry framework | N | N | Y | N | Y | Y | 10-week cycle | 4 asynchronous online modules + 3 synchronous coaching sessions (weeks 4, 7, 10) | Hybrid (online modules + synchronous web-based coaching) | External (trained research clinicians) |

| | | | | | | | | | | | | |
|---|---|---|---|---|---|---|---|---|---|---|---|---|
| Skåland et al. (2023) | Tuning in to Kids for Kindergarten Teachers (TIK-KT) | TIK-KT: training + fortnightly on-site supervision by a trained leader/pedagogue acting as a 'resource person' | N | Y | Y | N | N | N | Approximately 6 months (January-June 2020) | Initial training (1 day teachers / 2 days leaders) + fortnightly on-site supervision by the trained leader/pedagogue | In-person (group training + supervision at the kindergarten) | Internal (kindergarten leader/lead pedagogue, trained by external program trainers) |
| Spacciapoli et al. (2022) | Tools of the Mind | TREE: teacher video-based reflection + curricular-coach feedback (asynchronous model replacing repeated in-person visits) | N | N | Y | Y | Y | Y | 2-year professional-development period | Teacher-recorded classroom video submitted in periodic cycles throughout the school year | Asynchronous, video-based (digital platform) | External (Tools of the Mind curricular coach) |
| Stoiber et al. (2022) | PEBBLE (Positive and Evidence-Based Behavioral Learning Environment, a Tier I PBIS framework) | PEBBLE: consultation cycle with classroom-ecology observation + feedback + an individualized plan (IP) with goals | N | Y | Y | Y | Y | Y | 8 weeks | Baseline observation + consultation cycle with ongoing observations across 8 weeks | In-person, individual | External (15 trained consultants/coaches, also serving as observers) |
| Wang et al. (2024) | PEDALS (Positive Emotional Development and Learning Skills), based on Second Step Early Learning | PEDALS: coaches visit sites to provide implementation support at the classroom and site levels | N | N | N | N | Y | Y | 2-year program | Coaches visit sites 2x/month (year 1); coaching once a year (year 2) | In person | External (PEDALS coaches) |
| Wanless et al. (2013) | Responsive Classroom (RC) | Responsive Classroom (RC) - individualized coaching from the NEFC program | Y | N | N | N | N | Y | First and second year of RC implementation | Not quantified in number of sessions; ongoing individualized coaching throughout the school year | In-person, individual | External (certified RC/NEFC coaches) |

| | | | | | | | | | | | | |
|---|---|---|---|---|---|---|---|---|---|---|---|---|
| Welsh et al. (2024) | REDI (adapted for childcare centers, with the PATHS curriculum) | Adapted REDI: directors trained as coaches; personal-PD goal-setting + modeling videos + collaborative consultation | Y | N | N | Y | N | N | Not specified in number of weeks/months in the reviewed excerpt | Regular consultation meetings + individualized on-site coaching by the center director | In-person and hybrid (online modules + in-person coaching by the director) | Internal (center director, trained for the coaching role) |
| Welsh et al. (2025) | Head Start REDI | REDI: coach visits the classroom fortnightly to observe implementation and provide feedback and support | N | N | N | N | Y | Y | One school year | Initial in-person/online training + fortnightly coaching (12 expected visits) throughout the year | In-person and hybrid (online modules + in-person visits) | External (REDI coach with early-childhood and mentoring background, trained by REDI developers) |

*Table S3. SEL Program, coaching model, and active components (M = Modeling; PG = Guided Practice; R = Reflection; EM = Goal Setting; O = Observation; RD = Performance Feedback), duration, intensity, modality, and type of coach.*

## Table S3b. Format Structure and Measurement Instruments

| Study | Format Structure | Measurement Instruments |
|---|---|---|
| Arias de Sanchez & Li (2025) | Individualized Practice-Based Coaching (Snyder et al., 2015) | Semi-structured focus groups (3 waves, 2023-2025) |
| Berry et al. (2016) | Implementation support by PATHS coaches in consultation with teachers | SDQ (primary measure); PTRS; T-POT; semi-structured fidelity questionnaire |
| Bottiani et al. (2026) | Individualized, equity-focused coaching | Observational measures of student behavior; teacher self-report (incl. Racial Discomfort Scale); coaching contact logs |
| Cramer et al. (2021) | Individualized coaching (same model as Head Start CARES) | Teacher surveys; implementation-coach reports; classroom observation |
| Davies-Mercier et al. (2023) | Individualized consultation (ECCP consultant - teacher/provider) | Surveys of consultants (n=6), teachers (n=28) and parents (n=17); interviews; 2 focus groups |
| Domitrovich et al. (2010) | Individualized mentoring (REDI Trainers, lead+assistant teachers) | Monthly REDI Trainer Implementation Ratings (TIR); child emotion-recognition measures |
| Downer et al. (2018) | Individualized, video-based consultation | Validated measures of child engagement; teacher self-efficacy and attributions |
| Doyle et al. (2023) | Individualized video-based coaching (MyTeachingPartner) | CLASS (video segmentation); measures of hostile attribution bias and aggressive interpersonal negotiation; attendance; teacher-reported academic skills |
| Fox et al. (2011) | Individualized coaching (1 coach : 1 teacher) | TPOT; end-of-study social-validity questionnaire and interview |
| Franko et al. (2025) | Individualized consultation per Colorado's 'Essential Elements' model | Statewide interviews with multiple stakeholders |
| Garcia et al. (2022) | Individualized coaching by district-hired coaches | Not specified in the reviewed excerpt beyond student outcome and implementation measures |

| | | |
|---|---|---|
| Giordano et al. (2021) | Individualized coaching, comparing an internal (peer) coach vs. an external coach | Pyramid Model fidelity measure (e.g., TPOT); individual child social-skills assessment |
| Hardy et al. (2025) | Individualized coaching (Practice-Based Coaching) | Coaching Quality Checklist (CQC, 26 items, 3 subscales); TPOT |
| Harrington (2024) | Researcher-led PLC sessions functioning as coach/facilitator (CASEL-standard discussion + ESL-protocol modeling), followed by teacher-to-teacher peer classroom observation using a jointly-designed rubric | Researcher-created pre/post teacher self-evaluation surveys (Google Forms); session exit tickets; peer-observation walkthrough/rubric tool (non-evaluative); semi-structured interview protocols; no standardized/validated psychometric instrument |
| Hemmeter et al. (2015) | Individualized coaching (1 coach : 1 teacher) | TPOT (earlier version); team-designed checklists; CCBOT |
| Hemmeter et al. (2021) | Individualized coaching (Practice-Based Coaching) | TPOT; SSIS; DOCS; Mullen (direct child assessment) |
| Hunter et al. (2022) | Internal coaching (center director) + external support consultation (REDI consultants) | Classroom observation (delivery quality); teacher/director surveys; open-ended interview |
| Jennings et al. (2017) | Individual telephone coaching following group training | CLASS; self-report measures of emotion regulation, mindfulness, psychological distress, and time urgency (not specified by name in the reviewed excerpt) |
| Johnson (2019) | Individualized coaching by regional 'master cadre members' | TPOT |
| Kadik et al. (2025) | Collaborative classroom consultation (early childhood mental health consultant) | TPOT-SF (4 weeks after start and at the end); C-SDQ; DECA (child/family level) |
| Longhitano (2021) | Group lesson-study cycle facilitated by a mathematics instructional coach: collaborative planning meetings, PD sessions, classroom intervisitation (peer observation), and structured group debrief/reflection meetings | Researcher-created Likert-scale + open-ended teacher and student surveys (pre/post, based on Danielson's Framework for Teaching Domains 2–3); classroom observation forms; semi-structured interview protocols; no standardized/validated psychometric instrument |
| Mattera et al. (2013) | Individualized coaching (lead and assistant teacher jointly) | Weekly/monthly coaching logs; trainer fidelity records |
| Meyers et al. (2015) | Multi-level organizational consultation: external university-based/Special Services Unit consultants train teachers and provide building-level liaison support; from Year 3, transitions to a peer-based 'SEL champion' (stipended lead-teacher) model providing ongoing building-level modeling and support | No standardized/validated instrument reported. Implementation integrity checklists (self-report: lesson dates/length/components); individual and group stakeholder interviews; online administrator survey; teacher survey on implementation barriers |
| Morris et al. (2013) | Individualized in-classroom consultation (CCC) + a child-focused consultation component in the final third of the year | Observational ratings of teacher and child behavior; CSRP/FOL comparison measures |
| Partee et al. (2023) | Individualized consultation across 2 intensity tiers | TPOT-S; inCLASS (high-intensity tier); consultation logs; surveys; focus groups (n=20 participants) |
| Paxton C.L.C. et al. (2013) | Individualized coaching by certified NEFC coaches | CPOM; CPTS; CPFS; coach post-visit summaries |
| Rakap & Balikci (2025) | Individualized coaching | Target-practice checklist; child challenging-behavior measures |
| Robbie (2021) | Reciprocal peer-coaching dyads (no external/expert coach delivering direct coaching); each teacher alternately serves as both coach and coachee for their dyad partner | Direct behavioral observation via 15-minute video recordings, tallied for general praise, specific praise, non-specific corrective, and specific corrective statements; positive-to-negative interaction ratio calculated and tracked on a cumulative graph; end-of-study social-validity interview |
| Siller et al. (2024) | Individualized coaching | SRS-2; SSIS-SEL; study-developed coaching fidelity scale; classroom video coding |
| Skåland et al. (2023) | Group/individual supervision by trained leader (cascade model) | Semi-structured interviews (pre/post) |
| Spacciapoli et al. (2022) | Individualized, technology-mediated coaching (curricular coach) | TREE system (video-based fidelity measurement with indicators by focus area) |
| Stoiber et al. (2022) | Collaborative consultation (coach-teacher) | CPBO; PSIM; TSRS; Goal Attainment Scaling (GAS) |

| | | |
|---|---|---|
| Wang et al. (2024) | Individualized coaching at the classroom and site levels | Classroom observation; teacher/assistant/director surveys; focus groups/interviews |
| Wanless et al. (2013) | Individualized coaching | Focus groups (Study 1); multi-informant validation (Study 2) |
| Welsh et al. (2024) | Individualized coaching by the center director (internal trained coach) | Not specified in detail in the reviewed excerpt (instructional quality, emotion coaching, rich language) |
| Welsh et al. (2025) | Individualized coaching | Modified CLASS; dosage-fidelity and child-engagement logs; post-implementation qualitative interviews |

*Table S3b. Structure of the coaching delivery format and measurement tools used.*

# Table S4. Comparison conditions, reliability, and results

| Study | Comparison Condition | Reported Fidelity | Reported Dosage | Reported Quality | Student Outcomes | Outcome Type | Reported Effect Magnitude |
|---|---|---|---|---|---|---|---|
| Arias de Sanchez & Li (2025) | No comparison group (qualitative action research) | N | N | Y | N | Paradigm tensions (interpretivism vs. positivism) in the experience of PM implementation | N/A (qualitative) |
| Berry et al. (2016) | Control group (non-PATHS schools, random assignment) | Y (fidelity-questionnaire completion rate: 27% in year 1) | Y | Y | Y | Child behavioral and emotional difficulties (SDQ, primary); teacher-rated PATHS adherence (PTRS, secondary) | No significant differences on the SDQ at 1 and 2 years; significant differences on 6/11 PTRS subscales at 1 year |
| Bottiani et al. (2026) | Comparison with the standard Second Step (SEL) condition without the equity component | Y | Y | Y (teacher affect/engagement scores in coaching logs) | Y | Student physical aggression; general teacher self-efficacy; teacher racial discomfort | Significant effects on 1/6 observational measures (physical aggression) and 1/3 self-report measures (general self-efficacy) |
| Cramer et al. (2021) | No comparison group in this specific analysis | Y | N | N | N | Teacher implementation fidelity, predicted by commitment, stress, collegial support, and baseline classroom quality | Baseline commitment consistently predicted fidelity across time |
| Davies-Mercier et al. (2023) | No comparison group (descriptive pilot evaluation) | N | N | Y | N | Teacher/parent/director perceptions of facilitators and barriers to implementing the ECCP model | N/A (qualitative/descriptive evaluation; the authors caution about the small sample size) |
| Domitrovich et al. (2010) | Intervention vs. usual Head Start classrooms (in the original RCT; this article analyzes only intervention classrooms) | Y | N | Y | Y | Fidelity, generalization, and child engagement by curriculum component; child social-emotional/language/literacy outcomes | Moderate-to-strong correlations between implementation dimensions; implementation variation associated with social-emotional child outcomes, not language/literacy |
| Downer et al. (2018) | Control group (random assignment) | N | Y (fortnightly video frequency) | N | Y | Teacher use of social-emotional teaching strategies, self-efficacy and attributions; positive/negative child engagement | LOOK impacts reported on teacher strategies, self-efficacy, and child engagement (specific statistics not detailed in the reviewed excerpt) |

| | | | | | | | |
|---|---|---|---|---|---|---|---|
| Doyle et al. (2023) | 4Rs only (without the MTP component) as the comparison condition | Y | Y (8 fortnightly cycles) | Y | Y | Child hostile attribution bias and aggressive interpersonal negotiation; attendance; teacher-reported academic skills | Significantly lower levels of hostile attribution bias/aggression; higher attendance and academic skills in 4Rs+MTP vs. 4Rs only |
| Fox et al. (2011) | No comparison group; within-subject design (baseline vs. intervention) | Y | N | N | N (TPOT measures teacher implementation; no direct child outcomes in the reviewed excerpt) | Teacher implementation fidelity of the Teaching Pyramid Model (TPOT) | Functional relation between the PD intervention (workshops+materials+guides+coaching with feedback) and implementation; 2 of 3 teachers reached criterion |
| Franko et al. (2025) | No comparison group (statewide policy case study) | N | N | Y | N | Development of a statewide IECMHC model; identification of essential consultation elements | N/A (qualitative case study) |
| Garcia et al. (2022) | Business-as-usual | Y | Y | Y | Y | Teacher fidelity (TPOT); early childhood social-emotional and academic outcomes | Small, non-significant effects on math skills (PreK) and social problem-solving (K) |
| Giordano et al. (2021) | Comparison between two coach types (internal vs. external); no no-coaching group | Y | N | N | Y | Pyramid Model implementation fidelity; individual child social skills | Internal (peer) coaches more effective at supporting implementation fidelity; significant increase in social skills with internal coaching |
| Hardy et al. (2025) | Correlational analysis within the intervention group (no experimental comparison in this article) | Y | N | Y | N | Change in teacher social-emotional teaching practices (TPOT) in relation to coaching quality (CQC) | Total CQC significantly correlated with TPOT change; Change-Oriented subscale significantly correlated with growth; 82% of CQC variability attributable to the coach |
| Harrington (2024) | None; single-group, within-subject pre/post design across 2 sequential action-research cycles, no concurrent comparison group | N (no formal fidelity/adherence measure of PD or peer-observation delivery; exit tickets capture engagement/understanding, not fidelity to a protocol) | Y (PD series duration ~4 months; 5 peer observations of 30–45 min each explicitly quantified; full session schedule in Appendix A, Table A1) | N | N (outcomes measured are teacher self-reported perceptions/practice change; no direct/standardized child outcome measure administered, though the stated long-term aim is student SEL/achievement gain) | Teacher perceptions, knowledge, and self-reported implementation of SEL/ESL strategies (pre/post self-evaluation); qualitative themes on curriculum cultural-relevance, student participation, and instructional quality | N (only descriptive/graphical pre-post comparison reported for n=4 teachers; no inferential statistic or effect size computed, consistent with the small-N practitioner action-research design) |
| Hemmeter et al. (2015) | No comparison group; within-subject design (baseline vs. intervention) | Y | Y | N | Y | Teacher implementation fidelity (primary); classroom challenging behavior (CCBOT, secondary) | Functional relation established (9 demonstrations/replications); mixed generalization and maintenance |

| | | | | | | | |
|---|---|---|---|---|---|---|---|
| Hemmeter et al. (2021) | Business-as-usual (PD provided independently by the school system) | Y (29.29% of coaching sessions coded; 93.71% protocol agreement) | Y | Y | Y | Social-emotional, behavioral, and other developmental child outcomes (focal and non-focal) | Statistically significant and notable effects on social skills and challenging behavior (focal and non-focal) |
| Hunter et al. (2022) | Centers randomized to 'usual practice' in the larger study; this article analyzes only the intervention group | N (focus on delivery quality, not strict fidelity) | N | Y | N | REDI curriculum delivery quality and generalized use of teaching strategies | Teacher characteristics explained 40-45% of variance in delivery quality; work-environment factors 18-41% |
| Jennings et al. (2017) | Waitlist control group | Y (training fidelity coders) | Y | Y (CARE Facilitator Rating Form) | N | Teacher social-emotional competence (emotion regulation, mindfulness, distress, time urgency); classroom interaction quality (CLASS, emotional support) | Significant direct effects on adaptive emotion regulation, mindfulness, psychological distress, and time urgency; a protective effect on CLASS emotional support |
| Johnson (2019) | No control group; comparison across 7 early childhood program types | Y | Y | Y | N | Pyramid Model implementation fidelity (TPOT) by program type and region | Significant differences in fidelity by program type, both at baseline and in change following training and coaching |
| Kadik et al. (2025) | No control group (single-group pre-post design) | N | Y | N | Y | Teacher classroom/behavior-management practices; child protective factors and behavior problems | Significant pre-post improvements in classroom climate/management and in child protective factors/behavior problems (n=138 children assessed); larger gains among African American children |
| Longhitano (2021) | None; single-group pre/post design, no comparison group | N (no formal fidelity/adherence checklist for the lesson-study protocol itself is reported, beyond narrative description of meeting protocols being distributed and followed) | Y (specific components enumerated per teacher: 1 pre-interview, 1 pre-survey, PD session(s), planning meetings, 1 peer observation, 1 post-survey, 1 exit interview, over one school year) | N | Y, with an explicit author-acknowledged limitation, a student self-report survey (attitudes/self-efficacy in mathematics) was administered, but sample size was insufficient to draw robust conclusions for RQ3; findings for students are largely qualitative/anecdotal (e.g., teacher-reported case of a lab student passing the Algebra I Regents exam) | Teacher: self-rated DFT-domain practice change (Likert, pre/post) plus qualitative thematic findings on practice/perception change. Student: self-reported attitudes and mathematics self-efficacy (Likert, pre/post), explicitly underpowered per the author | CT: only descriptive, individual-level pre/post score changes reported (e.g., one teacher's mean DFT rating rose from 3.2 to 3.5; domain-specific increases/decreases reported per teacher); no aggregate inferential statistic or formal effect size, and results are mixed across the 5 teachers (2 declined) |

| | | | | | | | |
|---|---|---|---|---|---|---|---|
| Mattera et al. (2013) | Business-as-usual comparison group (random assignment in blocks of 4/8/12 centers) | Y | Y | Y (trainer rating of coach quality, 1-5 scale) | N (this report focuses on implementation; child outcomes are reported in a companion report) | Teacher fidelity and implementation of the 3 SEL enhancements; coaching quality and dosage | Moderate-to-large effect sizes on teacher practices (0.30 to 0.92, first-order) |
| Meyers et al. (2015) | A matched, randomly-assigned classroom control condition is described as planned for the Year-1 quasi-experimental evaluation; however, this article reports only the consultation PROCESS, and no comparative outcome results are presented | Y (implementation-integrity checklists tracking lesson dates/length/components; response rate explicitly reported as low in Year 1, improving in Year 2) | Y (lesson dosage explicitly reported: originally >70 lessons/year, revised to 24 core lessons/year, ~weekly; Special Services Unit staff present in buildings ~1 day/week) | N (no independent fidelity-quality rating instrument; only completion/checklist tracking) | N (article reports the consultation/implementation process only; no child outcome measures or results are presented in the reviewed text) | Consultation-process/implementation outcomes (checklist completion rates, teacher buy-in, adaptations across years); no child outcome data reported | N (no quantitative effect size or statistical outcome reported; purely a narrative/descriptive account of process changes, e.g., improved checklist completion after Year-2 adjustments, described without statistics) |
| Morris et al. (2013) | Usual-preschool comparison group (centers randomized to FOL or usual practice) | Y | Y | N | Y | Teacher classroom management; instructional time; child behavior and approaches to learning | Improvements in teacher classroom management in focal areas; greater instructional time in Newark; partial sustainability 1 year later |
| Partee et al. (2023) | No comparison group (pilot implementation study) | Y | Y | N | N (focus on implementation tensions, not direct child outcomes in this article) | Implementation tensions (plans vs. reality; deficit vs. strengths framing; systemic factors) | N/A (the article focuses on qualitative analysis of implementation tensions, not on effect sizes) |
| Paxton C.L.C. et al. (2013) | High- vs. low-fidelity comparison (not a classic control/intervention group) | Y | Y | Y | N | Teacher implementation fidelity (FOI) and associated coaching/school-context factors | d=5.14 in fidelity difference between high/low schools (t(4)=8.38, p=.001) |
| Rakap & Balikci (2025) | No comparison group; within-subject design (baseline vs. intervention) | Y | Y | N | Y | Teacher implementation fidelity (primary); child challenging behavior (secondary) | Functional relation demonstrated via visual analysis (not a traditional statistical effect size) |
| Robbie (2021) | No parallel control group; within-subject, non-concurrent multiple-baseline design in which each participant's own baseline phase serves as the comparison | Y (recording-completion fidelity explicitly quantified: Dyad 1 = 67% and 56% of target weekly recordings; Dyad 2 = 94%; author also documents specific fidelity threats, e.g., a participant receiving training while driving) | Y (baseline length, intervention-phase length in weeks, and faded-support duration explicitly reported per dyad) | N (no independent fidelity-quality rating beyond recording-completion percentages) | N (the dependent variable, positive-to-negative teacher/student interaction ratio, is a teacher behavioral outcome derived from teacher-student interaction counts, not a direct/standardized child outcome measure) | Teacher behavioral outcome: positive-to-negative teacher/student interaction ratio, derived from tallied praise/corrective statements during classroom observation | Y: low-to-moderate effect (TAU-U = 0.18, p = .02); no participant met PND effect criteria; 3 of 4 participants showed higher-than-baseline mean ratios during intervention; author explicitly concludes the intervention effect/functional relation was not strongly established given the small N and high data variability |
| Siller et al. (2024) | No comparison group (single-group design) | Y | Y | Y | Y (target child's active engagement, not broad SEL outcomes) | Program feasibility and acceptability; coaching fidelity; child active engagement (AE) | 9/10 feasibility benchmarks met; descriptive gains reported in active engagement |

| | | | | | | | |
|---|---|---|---|---|---|---|---|
| Skåland et al. (2023) | Qualitative sub-sample within a larger RCT with a comparison group (not applicable to this specific qualitative sub-sample) | Partial (implementation/motivation themes, not a formal fidelity measure) | Y (fortnightly supervision frequency) | N | N | Teacher/leader perceived motivation and implementation (research-to-practice gap) | N/A (qualitative) |
| Spacciapoli et al. (2022) | No comparison group (descriptive pilot) | Y | N | Y | N | Teacher implementation fidelity, longitudinal growth by focus area | Descriptive (growth visualized through an indicator 'tree,' no inferential statistics) |
| Stoiber et al. (2022) | No comparison group | Y | N | Y | N (focus on teacher practices; no direct child outcomes in the reviewed excerpt) | Positive/preventive classroom practices; change in instructional style and teacher affect | Moderate-to-strong pre-post changes (described qualitatively; no specific statistical effect size cited) |
| Wang et al. (2024) | Comparison group (similar classrooms without PEDALS, propensity-score weighting) | Y | Y | Y | Y (baseline data reported; impact results still pending) | Second Step curriculum implementation fidelity; child SEL skills (SSIS-SEL) and executive function (HTKS-R) | Baseline data presented; impact estimates not yet available in the reviewed implementation report |
| Wanless et al. (2013) | Qualitative sub-sample of a larger RCT (Responsive Classroom Efficacy Study) | Y (central theme: perceived fidelity associated with principal support and individualized coaching) | N | Y | N | Teacher/coach perceptions of factors influencing implementation fidelity | N/A (qualitative) |
| Welsh et al. (2024) | Comparison group (non-intervention centers), random assignment | Y (high teacher/director engagement and implementation reported) | N | Y | N (focus on teacher practices: responsive teaching, emotion coaching, rich language) | Teacher practices: responsive teaching, emotion coaching, use of rich language, instructional quality | Reported improvement in teacher practices vs. control (no specific effect size cited in the reviewed excerpt) |
| Welsh et al. (2025) | No comparison group (implementation pilot in a new context) | Y | Y | Y (teaching quality via modified CLASS) | N (focus on teacher fidelity/adaptation, not direct child outcomes) | Curriculum implementation fidelity; adaptations made by teachers; perceived usefulness | Descriptive (gradual improvement in sub-component implementation across the year) |

*Table S4. Stated comparison condition, implementation indicators (fidelity, dose, quality), reporting of student outcomes, and effect size.*

## Table S5. Theoretical Framework, Reflexivity, Funding, and Conflict of Interest

| Study | Theoretical Framework | Reflexivity Statement | Declared Funding | Declared Conflict of Interest |
|---|---|---|---|---|
| Arias de Sanchez & Li (2025) | Socio-constructivist and sociocultural theory; interpretivist/positivist paradigms | Y (professional reflexive processes highlighted as a central finding) | Y: Mitacs (ref #IT43383) and the Early Childhood Development Association | Y: 'The authors declare no conflicts of interest.' |
| Berry et al. (2016) | PATHS (Affective-Behavioral-Cognitive-Dynamic, ABCD, model) | N/A | Y (Birmingham City Council) | Y: 'Conflict of interest: None.' |

| | | | | |
|---|---|---|---|---|
| Bottiani et al. (2026) | Racial-equity-based violence-prevention framework | N/A | Y: NIMHD Grant R01MD013808 to the University of Virginia | Y, with a disclosed editorial-role note, no conflicts/competing interests declared; coauthor Catherine Bradshaw is editor of the publishing journal (Prevention Science), but peer review was managed by an independent associate editor |
| Cramer et al. (2021) | Theory of Planned Behavior; Health Action Process Approach | N/A | Y: IES Grant R305B140037 | Y: 'The authors declare that they have no conflict of interest.' |
| Davies-Mercier et al. (2023) | IECMHC framework | Y | Y (Maine Department of Health and Human Services, CCDF/ARPA funds) | Not reported |
| Domitrovich et al. (2010) | Domitrovich et al. (2008) framework | N/A | Y: NICHD Grants HD046064 and HD43763 awarded to Karen L. Bierman, Pennsylvania State University | Not reported |
| Downer et al. (2018) | LOOK model; theory of video-based reflective consultation | N/A | Y: IES Grant R305A120323-14 to the University of Virginia | Y, with a disclosed specific interest, Downer, Williford, and Bulotsky-Shearer were co-investigators on the IES grant that funded this study; all other authors declare no conflict of interest |
| Doyle et al. (2023) | MyTeachingPartner model; CLASS framework | N/A | Y: IES Grant R305A100590 to the University of Virginia | Y: 'The authors declare they have no financial or non-financial interests.' |
| Fox et al. (2011) | Teaching Pyramid Model | Y (end-of-study social-validity interview with teachers) | Y: U.S. Department of Education, Institute of Education Sciences, National Center for Special Education Research, Grant R324A07212 | Y: authors declared no potential conflicts of interest ('Declaration of Conflicting Interests') |
| Franko et al. (2025) | IECMHC framework; consultative stance (Johnston & Brinamen, 2006) | Y | Y: Preschool Development Grant Birth through Five Initiative (PDG B-5), Grant Number 90TP0054, Administration for Children and Families, U.S. DHHS | Y: 'The authors declare no conflicts of interest.' |
| Garcia et al. (2022) | Pyramid Model; scale-up framework for evidence-based practices | N/A | Y (U.S. Department of Education EIR grant) | Not reported |
| Giordano et al. (2021) | Practice-Based Coaching; PBIS | N/A | Not reported in the reviewed excerpt | Y: 'No potential conflict of interest was reported by the authors.' |
| Hardy et al. (2025) | Coaching Quality Framework | N/A | Y: IES Grant R324A120178 to Vanderbilt University | Y, with a disclosed specific interest, 'Mary Louise Hemmeter is an author of a book on the Pyramid Model, a book on Practice-Based Coaching, and the Teaching Pyramid Observation Tool; she receives royalties for all three publications' |
| Harrington (2024) | CASEL five-competency SEL framework; culturally responsive pedagogy; TESOL/ESL strategy frameworks; action-research/transformative-practice framework (Herr & Anderson, 2015) | Y: one of the most extensive reflexivity treatments in the corpus: a dedicated 'Positionality' section explicitly examining the researcher's triple positionality (Insider; Insider-collaborating-with-insiders; Outsider studying insiders, per Herr & Anderson, 2015) and its implications for bias and trust | Not reported in the reviewed excerpt (no external grant mentioned; Northeastern University doctoral capstone) | Not reported (no formal COI statement included; dissertation format) |
| Hemmeter et al. (2015) | Pyramid Model; Practice-Based Coaching; applied behavior analysis | N/A | Y (IES/NCSER Grant R324A070212) | Y (standard disclosure, no COI) |

| | | | | |
|---|---|---|---|---|
| Hemmeter et al. (2021) | Pyramid Model; Practice-Based Coaching | N/A | Y (IES/NCSER Grant R324A120178, Vanderbilt University) | Y, with a disclosed specific interest, 'Drs. Hemmeter, Fox, and Snyder are authors of the Teaching Pyramid Observation Tool and receive a portion of royalties' (the TPOT is the study's own fidelity/outcome measure) |
| Hunter et al. (2022) | REDI model; theory of teacher readiness/receptiveness to consultation | N/A | Y: NICHD Grant HD079410; IES Grant R305B090007; Society for the Study of School Psychology dissertation grant | Y: authors declare the research was conducted in the absence of any commercial or financial relationships that could be a potential conflict of interest |
| Jennings et al. (2017) | Mindfulness framework applied to teacher social-emotional competence | N/A | Y: IES Award Numbers R305A120180 and R305A140692 | Not reported |
| Johnson (2019) | PBIS; Pyramid Model; implementation science at scale | N/A | Y (Minnesota Department of Education, federal CFDA #84.181 funding) | Y: 'No potential conflict of interest was reported by the authors.' |
| Kadik et al. (2025) | IECMHC framework (consultative stance); three-tiered ecological theory (site, classroom, child/family) | N/A | Y (NYC Department of Health and Mental Hygiene) | Not reported |
| Longhitano (2021) | Danielson's Framework for Teaching (DFT); lesson study; transformational learning in adults (Cox, 2015); Bandura's (1977) self-efficacy theory; collective teacher efficacy; equity/de-tracking framework | N (no explicit researcher-positionality statement identified in the reviewed excerpt, despite the researcher's dual role as instructional coach and investigator) | Not reported in the reviewed excerpt | Not reported in the reviewed excerpt |
| Mattera et al. (2013) | Program-specific theories of change (Incredible Years, PATHS, Tools of the Mind) | N/A | Y (demonstration funded via MDRC/OPRE; study funds for hiring coaches) | Not reported |
| Meyers et al. (2015) | Ecological model of organizational consultation (Bronfenbrenner, Lewin, Schein, Vygotsky) | N (no explicit statement of researcher positionality/reflexivity, despite co-authors holding dual roles as consultants/administrators and Project Evaluators) | Yes: Illinois Children's Healthcare Foundation (ILCHF) grant (2010) to the Livingston County Children's Network, supporting a 16-month planning period plus 3 years of implementation | Yes: authors report no financial relationships or affiliations that could bias the work ('to the best of their knowledge... no financial relationships or affiliations that could influence or bias the opinions, decisions, or work presented') |
| Morris et al. (2013) | The Incredible Years Teaching Pyramid | N/A | Y: philanthropic funder consortium: The George Gund Foundation, The Grable Foundation, The John D. and Catherine T. MacArthur Foundation, The Joyce Foundation, The Kresge Foundation, McCormick Foundation, The Nicholson Foundation, The Pew Charitable Trusts, Robert Wood Johnson Foundation | Not reported |
| Partee et al. (2023) | Domitrovich et al. (2008) implementation framework | Y | Y: Virginia Department of Education, via federal Governor's Emergency Education Relief (GEER) Fund | Y: authors declare the research was conducted in the absence of any commercial or financial relationships that could be a potential conflict of interest |
| Paxton C.L.C. et al. (2013) | Domitrovich et al. (2008) implementation fidelity framework | Y | Y (IES Grants R305B090002 and R305A070063, University of Virginia) | Not reported |

| | | | | |
|---|---|---|---|---|
| Rakap & Balikci (2025) | Pyramid Model; Practice-Based Coaching | N/A | Y: study received no specific research grant; open-access publication fee funded by TÜBİTAK (Scientific and Technological Research Council of Türkiye) | Y: 'The authors declare that they have no conflicts of interest.' |
| Robbie (2021) | Applied behavior analysis; evidence-based classroom management (EBCM)/PBIS; Darling-Hammond et al. (2017) core features of effective teacher PD; habit-formation theory (Duhigg, 2012) | Y (end-of-study social-validity interviews explicitly probed and reported participant experience, including self-identified threats to fidelity/internal validity, e.g., participants admitting to watching their own videos) | Not reported (no external grant disclosed; doctoral dissertation research) | Not reported (no formal COI statement included; dissertation format) |
| Siller et al. (2024) | Active Engagement transdiagnostic model; Appreciative Inquiry | N/A | Y: NICHD Grant R21HD100820; The Marcus Foundation; Children's Research Trust | Not reported |
| Skåland et al. (2023) | Theory of Planned Behavior; attachment/emotion-regulation theory (TIK) | Y | Y: 'This research received no specific grant from any funding agency in the public, commercial, or not-for-profit sectors'; open-access fee funded by University of Oslo | Not reported |
| Spacciapoli et al. (2022) | Vygotskian sociocultural theory; implementation fidelity framework | N/A | Not reported in the reviewed excerpt | Y: authors declare the research was conducted in the absence of any commercial or financial relationships that could be a potential conflict of interest |
| Stoiber et al. (2022) | Tier I PBIS; collaborative consultation theory | Y (direct teacher quote about the reflective-practice component) | Not reported in the reviewed excerpt | Y: 'No potential conflict of interest was reported by the author(s).' |
| Wang et al. (2024) | PEDALS model of integrated supports for SEL implementation in PreK | N/A | Y: Ralph C. Wilson, Jr. Foundation and the Health Foundation for Western and Central New York | CT: no per-article COI statement; RAND cites its general institutional research-integrity policy (staff training, project screening, mandatory disclosure) rather than a study-specific declaration |
| Wanless et al. (2013) | Domitrovich et al. framework on multi-level implementation fidelity | Y | Y: IES Grant R305A070063 to the University of Virginia | Not reported |
| Welsh et al. (2024) | REDI model; teacher agency theory in professional development | N/A | Y: NICHD Grant HD079410 | Not reported |
| Welsh et al. (2025) | REDI framework; cultural adaptation of evidence-based interventions | Y (qualitative interviews reflecting teachers' views on necessary cultural adjustments) | Y: Annie E. Casey Foundation | Y: 'All authors report no conflict of interest, financial [or otherwise].' |

*Table S5. Explicit theoretical framework, presence of a statement of reflexivity (applicable primarily to qualitative and mixed-methods designs), and statements regarding funding and conflicts of interest, verified against the full text of each study.*

# Table S6. MMAT Categorization Scores

| Study | MMAT Category | Categorization Note |
| --- | --- | --- |
| Arias de Sanchez & Li (2025) | 1 (Qualitative) | 1 (Qualitative) |
| Berry et al. (2016) | 2 (RCT) | 2 (RCT) |
| Bottiani et al. (2026) | 2 (RCT) | 2 (RCT) |
| Cramer et al. (2021) | 4 (Quantitative descriptive) | 4 (Quantitative descriptive) |
| Davies-Mercier et al. (2023) | 5 (Mixed) / 4+1 | 5 (Mixed methods) + 4 (quant component) + 1 (qual component) |
| Domitrovich et al. (2010) | 4 (Quantitative descriptive) | 4 (Quantitative descriptive) |
| Downer et al. (2018) | 2 (RCT) | 2 (RCT) |
| Doyle et al. (2023) | 3 (Quantitative nonrandomized) | 3 (Quantitative nonrandomized) |
| Fox et al. (2011) | 5 (Mixed) / 3+1 | 5 (Mixed methods) + 3 (quant component) + 1 (qual component) |
| Franko et al. (2025) | 1 (Qualitative) | 1 (Qualitative) |
| Garcia et al. (2022) | 2 (RCT) | 2 (RCT) |
| Giordano et al. (2021) | 3 (Quantitative nonrandomized) | 3 (Quantitative nonrandomized) |
| Hardy et al. (2025) | 4 (Quantitative descriptive) | 4 (Quantitative descriptive) |
| Harrington (2024) | 5 (Mixed) / 1+4 | 5 (Mixed methods, embedded) + 1 (qualitative action research, dominant strand) + 4 (quantitative descriptive, embedded: pre/post self-evaluation surveys, n=4 teachers, no inferential statistics) |
| Hemmeter et al. (2015) | 3 (Quantitative nonrandomized) | 3 (Quantitative nonrandomized) |
| Hemmeter et al. (2021) | 2 (RCT) | 2 (RCT) |
| Hunter et al. (2022) | 5 (Mixed) / 4+1 | 5 (Mixed methods) + 4 (quant component) + 1 (qual component) |
| Jennings et al. (2017) | 2 (RCT) | 2 (RCT) |
| Johnson (2019) | 4 (Quantitative descriptive) | 4 (Quantitative descriptive) |
| Kadik et al. (2025) | 3 (Quantitative nonrandomized) | 3 (Quantitative nonrandomized) |
| Longhitano (2021) | 5 (Mixed) / 1+4 | 5 (Mixed methods, embedded) + 1 (qualitative lesson-study case analysis, dominant) + 4 (quantitative descriptive, embedded: pre/post Likert teacher and student self-ratings, n=5 teachers, explicitly underpowered student component) |
| Mattera et al. (2013) | 2 (RCT) | 2 (RCT) |
| Meyers et al. (2015) | 1 (Qualitative) | 1 (Qualitative): case study / formative program evaluation of a systems-level organizational-consultation model; no quantitative or mixed-methods component (the planned Year-1 quasi-experimental outcome evaluation is not reported in this article) |
| Morris et al. (2013) | 2 (RCT) | 2 (RCT) |
| Partee et al. (2023) | 5 (Mixed) / 4+1 | 5 (Mixed methods) + 4 (quant component) + 1 (qual component) |
| Paxton C.L.C. et al. (2013) | 5 (Mixed) / 4+1 | 5 (Mixed methods) + 4 (quant component) + 1 (qual component) |
| Rakap & Balikci (2025) | 3 (Quantitative nonrandomized) | 3 (Quantitative nonrandomized) |

| | | |
|---|---|---|
| Robbie (2021) | 5 (Mixed) / 3+1 | 5 (Mixed methods) + 3 (quant component: non-concurrent single-case multiple-baseline, predominant) + 1 (qual component: end-of-study social-validity interviews, minor) |
| Siller et al. (2024) | 3 (Quantitative nonrandomized) | 3 (Quantitative nonrandomized) |
| Skåland et al. (2023) | 1 (Qualitative) | 1 (Qualitative) |
| Spacciapoli et al. (2022) | 4 (Quantitative descriptive) | 4 (Quantitative descriptive) |
| Stoiber et al. (2022) | 3 (Quantitative nonrandomized) | 3 (Quantitative nonrandomized) |
| Wang et al. (2024) | 5 (Mixed) / 3+1 | 5 (Mixed methods) + 3 (quant component) + 1 (qual component) |
| Wanless et al. (2013) | 1 (Qualitative) | 1 (Qualitative) |
| Welsh et al. (2024) | 2 (RCT) | 2 (RCT) |
| Welsh et al. (2025) | 5 (Mixed) / 4+1 | 5 (Mixed methods) + 4 (quant component) + 1 (qual component) |

*Table S6. MMAT design category assigned to each study and rationale for the categorization, including the composition of the mixed-methods studies (qualitative component + quantitative component).*

## Table S7. Summary of Qualitative Findings (GRADE-CERQual)

GRADE-CERQual assessment of the qualitative-strand findings synthesized in Section 3.3 of the main manuscript, following Lewin et al. (2018). Contributing studies are restricted to the 15 of 36 included studies with a qualitative component (5 purely qualitative + the qualitative component of 10 mixed-methods studies; see Table S6). Confidence in the quantitative strand was not formally graded, consistent with the review's non-meta-analytic synthesis design (manuscript Section 2.7.2).

| Review Finding | Contributing Studies | Methodological Limitations | Coherence | Adequacy of Data | Relevance | Overall CERQual Confidence |
|---|---|---|---|---|---|---|
| School leadership support, protected time, and individual teacher disposition jointly shape teachers' uptake of coaching within the school's inner and outer setting. | Wanless et al. (2013); Hunter et al. (2022); Harrington (2024); Robbie (2021) | Moderate concerns: qualitative evidence is drawn mainly from qualitative sub-samples embedded in larger quantitative studies or a small-N practitioner action-research dissertation; none used member-checking or triangulated coding. | No or very minor concerns: consistent pattern across four studies in different U.S. contexts. | No concerns: four studies contributing richly described, convergent qualitative data. | Moderate concerns: all four studies are U.S.-based; applicability to lower-resource or non-U.S. systems is inferred rather than demonstrated. | Moderate |
| Coaches shift, at least temporarily, from technical-pedagogical assistance toward providing socioemotional support for teachers themselves under conditions of high stress or systemic disruption. | Partee et al. (2023); Harrington (2024) | Serious concerns: based on only two studies; one reports this as a secondary qualitative theme rather than a primary research question. | No concerns: both studies converge on the same mechanism. | Serious concerns: thin qualitative data, limited depth of description across only two sources. | Minor concerns: both studies address high-need, resource-constrained U.S. school settings, which increases relevance to lower-resource contexts. | Low |
| Effective coaching combines a structured technical cycle (observation, modeling, feedback, goal setting) with a trust-based, explicitly non-evaluative consultative alliance; when coaching is perceived as evaluative, active ingredients fail to translate into practice change. | Fox et al. (2011); Arias de Sanchez & Li (2025); Franko et al. (2025); Robbie (2021) | Moderate concerns: mix of single-case, action-research, and case-study designs with limited use of formal qualitative rigor procedures. | No or very minor concerns: consistent across four studies spanning different coaching models (Pyramid Model, IECMHC, peer coaching). | No concerns: four studies with substantive qualitative or mixed-methods detail on the coach–teacher relationship. | Moderate concerns: three of four studies are U.S.-based; one is Canadian; equity/Global South applicability is inferred. | Moderate |

| | | | | | | |
|---|---|---|---|---|---|---|
| Scaling manualized SEL programs into real school systems surfaces a tension between fidelity to the intervention manual and the need for culturally responsive adaptation, with explicit equity implications for historically marginalized students. | Arias de Sanchez & Li (2025); Partee et al. (2023); Welsh et al. (2025) | Moderate concerns: qualitative components are secondary to the primary (often quantitative or mixed) aims of two of the three contributing studies. | No or very minor concerns: all three studies converge on the same fidelity-adaptation-equity tension. | Minor concerns: three studies with moderately rich contextual detail, but none was designed primarily to examine equity as its central research question. | Minor concerns: this finding directly addresses cultural adaptation and equity, which increases its relevance to the review's stated interest in the Global South and underrepresented contexts. | Moderate |

## Table S8. Item-Level MMAT 2018 Risk-of-Bias Ratings by Design Category

Item-level Mixed Methods Appraisal Tool (MMAT 2018; Hong et al., 2018) ratings (Yes / No / Can't tell / N/A) for each of the 36 included studies, organized by MMAT design category. Ratings shown are the consensus rating reached by the two independent appraisers (A.P.C., C.V.C.C.; Section 2.6). S1 and S2 are the two screening questions applied to every study regardless of category. Mixed-methods studies contribute their qualitative-component ratings to Table S8a, their quantitative-component ratings to Table S8c or S8d as applicable, and their five mixed-methods-specific ratings to Table S8e.

## Table S8a. Qualitative-Component Ratings (Category 1 criteria)

Table S8a. Applies to the 5 exclusively qualitative studies and to the qualitative component of the 10 mixed-methods studies (n = 15).

| Study | S1 | S2 | 1.1 Approach appropriate | 1.2 Data collection adequate | 1.3 Findings derived from data | 1.4 Interpretation substantiated | 1.5 Coherence sources/analysis |
|---|---|---|---|---|---|---|---|
| Fox et al. (2011) | Y | Y | Y | CT | CT | Y | CT |
| Hunter et al. (2022) | Y | Y | Y | Y | Y | Y | Y |
| Skåland et al. (2023) | Y | Y | Y | Y | Y | Y | Y |
| Meyers et al. (2015) | Y | Y | Y | CT | CT | CT | Y |
| Wanless et al. (2013) | Y | Y | Y | Y | Y | CT | Y |
| Arias de Sanchez & Li (2025) | Y | Y | Y | Y | Y | Y | Y |
| Partee et al. (2023) | Y | Y | Y | Y | Y | Y | Y |
| Harrington (2024) | Y | Y | Y | Y | CT | CT | Y |
| Welsh et al. (2025) | Y | Y | Y | Y | Y | Y | Y |
| Wang et al. (2024) | Y | Y | Y | Y | Y | Y | Y |
| Franko et al. (2025) | Y | Y | Y | Y | CT | CT | CT |
| Longhitano (2021) | Y | Y | Y | Y | Y | Y | Y |

| | | | | | | | |
|---|---|---|---|---|---|---|---|
| Davies-Mercier et al. (2023) | Y | Y | Y | Y | Y | Y | Y |
| Paxton et al. (2013) | Y | Y | Y | Y | Y | Y | Y |
| Robbie (2021) | Y | Y | Y | CT | Y | Y | Y |

# Table S8b. Randomized Controlled Trial Ratings (Category 2 criteria)

Table S8b. Applies to the 9 randomized controlled trials (n = 9).

| **Study** | **S1** | **S2** | **2.1 Randomization appropriate** | **2.2 Groups comparable at baseline** | **2.3 Complete outcome data** | **2.4 Assessors blinded** | **2.5 Adherence to intervention** |
|---|---|---|---|---|---|---|---|
| Berry et al. (2016) | Y | Y | Y | Y | Y | CT | Y |
| Downer et al. (2018) | Y | Y | Y | CT | CT | CT | Y |
| Hemmeter et al. (2021) | Y | Y | Y | CT | Y | CT | Y |
| Bottiani et al. (2026) | Y | Y | Y | CT | CT | CT | Y |
| Welsh et al. (2024) | Y | Y | Y | CT | CT | CT | Y |
| Garcia et al. (2022) | Y | Y | Y | CT | CT | CT | Y |
| Jennings et al. (2017) | Y | Y | Y | CT | Y | CT | Y |
| Mattera et al. (2013) | Y | Y | Y | CT | CT | CT | Y |
| Morris et al. (2013) | Y | Y | Y | CT | Y | CT | Y |

# Table S8c. Non-Randomized Quantitative Ratings (Category 3 criteria)

Table S8c. Applies to the 7 exclusively non-randomized quantitative studies and to the quantitative component of the 3 mixed-methods studies combining a qualitative and a non-randomized quantitative component (n = 10).

| **Study** | **S1** | **S2** | **3.1 Participants representative** | **3.2 Measurements appropriate** | **3.3 Complete outcome data** | **3.4 Confounders accounted for** | **3.5 Intervention administered as intended** |
|---|---|---|---|---|---|---|---|
| Fox et al. (2011) | Y | Y | N | Y | CT | N | Y |
| Rakap & Balikci (2025) | Y | Y | N | Y | CT | N | Y |
| Stoiber et al. (2022) | Y | Y | CT | Y | CT | N | Y |
| Siller et al. (2024) | Y | Y | CT | Y | CT | N | Y |

| | | | | | | | |
|---|---|---|---|---|---|---|---|
| Giordano et al. (2021) | Y | Y | N | Y | CT | N | Y |
| Doyle et al. (2023) | Y | Y | CT | Y | CT | CT | Y |
| Wang et al. (2024) | Y | Y | CT | Y | CT | CT | Y |
| Kadik et al. (2025) | Y | Y | Y | Y | CT | N | Y |
| Hemmeter et al. (2015) | Y | Y | N | Y | Y | N | Y |
| Robbie (2021) | Y | Y | N | Y | CT | CT | N |

## Table S8d. Descriptive Quantitative Ratings (Category 4 criteria)

Table S8d. Applies to the 5 exclusively descriptive quantitative studies and to the quantitative component of the 7 mixed-methods studies combining a qualitative and a descriptive quantitative component (n = 12).

| **Study** | **S1** | **S2** | **4.1 Sampling strategy relevant** | **4.2 Sample representative** | **4.3 Measurements appropriate** | **4.4 Low non-response bias** | **4.5 Statistical analysis appropriate** |
|---|---|---|---|---|---|---|---|
| Spacciapoli et al. (2022) | Y | Y | Y | CT | Y | CT | Y |
| Hunter et al. (2022) | Y | Y | Y | CT | Y | CT | Y |
| Johnson (2019) | Y | Y | Y | CT | Y | CT | Y |
| Domitrovich et al. (2010) | Y | Y | Y | CT | Y | CT | Y |
| Partee et al. (2023) | Y | Y | Y | CT | Y | CT | Y |
| Cramer et al. (2021) | Y | Y | Y | Y | Y | CT | Y |
| Hardy et al. (2025) | Y | Y | Y | CT | Y | CT | Y |
| Harrington (2024) | Y | Y | Y | N | CT | CT | CT |
| Welsh et al. (2025) | Y | Y | Y | CT | Y | CT | Y |
| Longhitano (2021) | Y | Y | Y | N | CT | N | N |
| Davies-Mercier et al. (2023) | Y | Y | Y | N | Y | CT | Y |
| Paxton et al. (2013) | Y | Y | Y | CT | Y | CT | Y |

# Table S8e. Mixed-Methods-Specific Ratings (Category 5 criteria)

Table S8e. Applies to the 10 mixed-methods studies (component combination noted per study: quantitative-descriptive + qualitative, or quantitative-non-randomized + qualitative).

| Study | S1 | S2 | 5.1 Rationale for mixed design | 5.2 Components integrated | 5.3 Integration interpreted | 5.4 Divergences addressed | 5.5 Adherence to each tradition |
|---|---|---|---|---|---|---|---|
| Fox et al. (2011) | Y | Y | CT | CT | CT | CT | Y |
| Hunter et al. (2022) | Y | Y | Y | CT | CT | CT | Y |
| Partee et al. (2023) | Y | Y | Y | CT | CT | CT | Y |
| Harrington (2024) | Y | Y | CT | Y | Y | CT | CT |
| Welsh et al. (2025) | Y | Y | Y | CT | CT | CT | Y |
| Wang et al. (2024) | Y | Y | Y | CT | CT | CT | Y |
| Longhitano (2021) | Y | Y | Y | Y | Y | Y | CT |
| Davies-Mercier et al. (2023) | Y | Y | Y | CT | CT | CT | Y |
| Paxton et al. (2013) | Y | Y | Y | CT | CT | CT | Y |
| Robbie (2021) | Y | Y | CT | Y | Y | CT | CT |

# Table S9. Borderline Records Excluded at Full-Text Screening after Initial Reviewer Disagreement

Of the 20 full-text records on which the two independent reviewers (R1, R2) initially disagreed and which were referred to arbitration (J.D.D.C.; Section 2.4), 14 had appeared, on initial screening, to meet the eligibility criteria but were ultimately excluded after closer full-text examination (PRISMA 2020 item 16b; Page et al., 2021). They are listed here individually with their final exclusion code and the arbitrator's rationale.

| Study | Title | Final Exclusion Code | Reason for Exclusion |
|---|---|---|---|
| Nemiro et al. (2022) | Mental health and psychosocial wellbeing in education: The case to integrate core actions and interventions into learning environments | E4 | Secondary/conceptual review article; not a primary empirical study of teacher coaching/consultation with active components. |
| Clark et al. (2023) | Profiles of teachers' classroom management style: Differences in perceived school climate and professional characteristics | E1/E2 | Purely correlational/cross-sectional survey study; no coaching/consultation intervention present; sample includes upper-secondary (ISCED 3) teachers. |
| Bølstad et al. (2023) | Emotional competence training promotes teachers' emotion socialization and classroom environment: Effects from a TIK-in-School pilot study | E2 | Professional development consists entirely of 1.5-hour group training sessions; lacks a situated, individualized coaching/consultation component with in-classroom observation and active performance feedback. |
| Michalec & Wilson (2021) | Truth Hidden in Plain Sight: How Social–Emotional Learning Empowers Novice Teachers' Culturally Responsive Pedagogy in Title I Schools | E2 | Interview study on novice teachers' professional development; does not evaluate a structured coaching model. |
| Murray et al. (2025) | Operationalizing mindful co-regulation to build understanding of educator social-emotional competency | E5 | Measurement-validation study for a new observational instrument (MCRO); does not empirically evaluate outcomes/impact of the coaching intervention itself. |
| Ulla & Poom-Valickis (2023) | Program support matters: A systematic review on teacher- and school-related contextual factors facilitating the implementation of social-emotional learning programs | E4 | Secondary research (systematic literature review), not a primary empirical evaluation of a specific coaching/consultation intervention. |
| Yoder & Nolan (2018) | What Does SEL Look Like in the Classroom? | E4 | Practitioner-oriented article introducing an observation rubric; no research design, participant sample, or primary empirical data. |
| Afkinich et al. (2021) | “Needed Now More than Ever:” Infant and Early Childhood Mental Health Consultation in an Unprecedented Time | E2 | Descriptive study of pandemic-era service-delivery adaptation; does not empirically evaluate a structured teacher coaching/consultation intervention or measure teacher/student outcomes. |
| McDoniel et al. (2022) | Widely Used Measures of Classroom Quality Are Largely Unrelated to Preschool Skill Development | E2 | Observational analysis of associations between classroom-quality measures and preschool skill development; does not implement or evaluate a coaching/consultation intervention. |
| Schwartz & Dusenbury (2018) | District Recommendations for How States Can Support SEL | E4 | Policy guidance/recommendations document; no empirical research design, participants, or data collection. |
| Cho et al. (2021) | Happy Schools: Capacity Building for Learner Well-Being in the Asia-Pacific. Findings from the 2018-2020 Pilots in Japan, Lao PDR and Thailand | E2 | Broad capacity-building/wellbeing pilot programming; does not describe teacher coaching/consultation with the active components defined in the protocol. |
| Egan et al. (2019) | Elementary School Teachers' Preferences for School-Based Interventions for Students with Emotional and Behavioral Problems | E2 | Discrete choice experiment; teachers evaluate hypothetical intervention packages. No teacher received actual coaching; a preference study, not an intervention. |
| Carleton et al. (2021) | Renfrewshire's Nurturing Relationships Approach: Utilising Nurturing Approaches to Support School Staff and Pupils during COVID-19 | E1/E2 | Whole-school initiative whose core group mixes teachers with non-teaching staff (fails population criterion); narrative account of digital-resource development, not an individualized teacher coaching intervention with active components. |
| Barry et al. (2017) | Promoting Social and Emotional Well-Being in Schools | E4 | Critical perspective/literature review with preliminary case-study illustrations; secondary research, not a primary empirical evaluation of a specific coaching intervention. |

# Table S10. GRADE-Informed Certainty Assessment of Key Quantitative Findings

Certainty ratings for five key quantitative findings from the review, using a simplified adaptation of the GRADE domains (risk of bias, inconsistency, indirectness, imprecision, reporting bias; Guyatt et al., 2011) for narrative synthesis without meta-analysis (Section 2.7.2), complementing the GRADE-CERQual assessment of the qualitative strand in Table S7.

| Finding | Contributing Studies | Risk of Bias | Inconsistency | Indirectness | Imprecision | Reporting Bias | Overall Certainty |
|---|---|---|---|---|---|---|---|
| Structured, active-ingredient coaching cycles (observation, modeling, feedback, goal setting) increase teacher implementation fidelity relative to training-only conditions. | Fox et al. (2011); Hemmeter et al. (2015, 2021); Rakap & Balikci (2025); Berry et al. (2016) | Not serious | Not serious | Serious (mostly U.S./early-childhood contexts) | Not serious | Not serious | Moderate |
| Short-term effects of coaching on independently or standardized-assessed student academic outcomes are null or marginal. | Berry et al. (2016); Hemmeter et al. (2021); Morris et al. (2013); Garcia et al. (2022) | Not serious | Not serious (once outcome-reporter type is accounted for; Section 3.2) | Not serious | Serious (small samples in several contributing studies) | Not serious | Moderate |
| Fully asynchronous, video-mediated coaching formats are associated with lower teacher adherence/engagement than in-person or blended formats. | Welsh et al. (2024, 2025) | Serious | Cannot assess (too few studies) | Serious (single research group/context) | Very serious (only 2 studies) | Not serious | Very low |
| Internal (peer) coaching and external coaching present a trade-off (adherence/trust vs. technical rigor/role conflict) rather than a uniform advantage for either model. | Giordano et al. (2021); Robbie (2021); Franko et al. (2025) | Serious | Serious | Not serious | Serious (few high-rigor studies; Section 3.2) | Not serious | Low |
| An explicit equity/implicit-bias coaching supplement (R-CITY) is associated with reduced hostile-attribution/aggression and increased teacher self-efficacy relative to standard SEL coaching. | Bottiani et al. (2026) | Not serious | Cannot assess (single study) | Not serious | Serious (single trial; significant on only 1/6 and 1/3 measures) | Not serious | Low |

# Table S11. Full Database Search Strategies (Scopus, Web of Science, ERIC)

Verbatim Boolean search strings executed in each database on 22-23 June 2026, reported in accordance with PRISMA-S (Rethlefsen et al., 2021). Technical notes on adapting the canonical string to ERIC's native syntax: (i) no field-tag prefix is used, since the ERIC interface searches by default in title, abstract, and descriptors; (ii) in-phrase truncation ("emotion* coaching") was replaced with explicit lexical variants ("emotional coaching" OR "emotion coaching") because of a known limitation of the ERIC engine with asterisks inside quotation marks; (iii) both British and American spellings of behavioral/behavioural were preserved, since the ERIC native interface does not normalize them automatically; the "Peer reviewed only" filter was applied manually in the interface.

| Database | Field / Notes | Verbatim Search String | Execution Date | Raw Records Retrieved |
|---|---|---|---|---|
| Scopus (Elsevier) | TITLE-ABS-KEY field | TITLE-ABS-KEY(("social-emotional learning" OR SEL OR "social emotional learning" OR "social-emotional competenc*" OR "social emotional competenc*" OR "social-emotional teaching" OR "emotion* coaching" OR "school-based SEL") AND (teacher* OR educator* OR "school staff" OR classroom*) AND (coach* OR consultation OR consult* OR "practice-based coaching" OR "instructional coaching" OR "performance feedback" OR modeling OR modelling OR "guided reflection" OR "goal setting" OR observation OR "guided practice") AND ("implementation fidelity" OR fidelity OR implementation OR dosage OR adherence OR quality OR "program implementation" OR "implementation quality" OR "student outcome*" OR "academic outcome*" OR "behavior* outcome*" OR "social-emotional outcome*") AND (school* OR preschool* OR kindergarten OR "early childhood" OR elementary OR "middle school" OR "secondary school") NOT ("pre-service teacher*" OR "teacher candidate*" OR universit* OR college*)) | 22-23 June 2026 | 92 |
| Web of Science Core Collection (Clarivate) | Topic field (TS=); SSCI and ESCI indexes | TS=("social-emotional learning" OR SEL OR "social emotional learning" OR "social-emotional competenc*" OR "social emotional competenc*" OR "social-emotional teaching" OR "emotion* coaching" OR "school-based SEL") AND (teacher* OR educator* OR "school staff" OR classroom*) AND (coach* OR consultation OR consult* OR "practice-based coaching" OR "instructional coaching" OR "performance feedback" OR modeling OR modelling OR "guided reflection" OR "goal setting" OR observation OR "guided practice") AND ("implementation fidelity" OR fidelity OR implementation OR dosage OR adherence OR quality OR "program implementation" OR "implementation quality" OR "student outcome*" OR "academic outcome*" OR "behavior* outcome*" OR "social-emotional outcome*") AND (school* OR preschool* OR kindergarten OR "early childhood" OR elementary OR "middle school" OR "secondary school") NOT ("pre-service teacher*" OR "teacher candidate*" OR universit* OR college*) | 22-23 June 2026 | 123 |
| ERIC (Institute of Education Sciences) | Native eric.ed.gov interface; "Peer reviewed only" filter applied manually | ("social-emotional learning" OR SEL OR "social emotional learning" OR "social-emotional competence*" OR "social emotional competence*" OR "social-emotional teaching" OR "emotional coaching" OR "emotion coaching" OR "school-based SEL") AND (teacher* OR educator* OR "school staff" OR classroom*) AND (coach* OR consultation OR consult* OR "practice-based coaching" OR "instructional coaching" OR "performance feedback" OR modeling OR modelling OR "guided reflection" OR "goal setting" OR observation OR "guided practice") AND ("implementation fidelity" OR fidelity OR implementation OR dosage OR adherence OR quality OR "program implementation" OR "implementation quality" OR "student outcome*" OR "academic outcome*" OR "behavioral outcome*" OR "behavioural outcome*" OR "social-emotional outcome*") AND (school* OR preschool* OR kindergarten OR "early childhood" OR elementary OR "middle school" OR "secondary school") NOT ("pre-service teacher*" OR "teacher candidate*" OR universit* OR college*) | 22-23 June 2026 | 199 |

# Table S12. Protocol Amendments Documented and Justified in the Registered Protocol Text

This table reproduces the amendments that are explicitly documented, dated (by version number), and justified within the text of the registered protocol (version 1.7; OSF project M3846; Protocol_PRISMA-P_OSF_EN_v1.7). It is not a full reconstruction of the complete seven-round amendment log (v1.1-v1.7); the complete, dated, version-by-version log is maintained in the OSF project wiki, referenced in Section 2.1 of the manuscript.

| Version | Section Amended | Amendment | Justification (as stated in the protocol) |
|---|---|---|---|
| v1.2 | Eligibility — Population (protocol §8b) | Clarified that early childhood and preschool settings (e.g., Pyramid Model studies) are eligible under the ISCED 0-2 population criterion. | Made explicit to avoid reviewer ambiguity, consistent with the original protocol wording. |
| v1.4 | Eligibility — Language (protocol §8a) | Removed the original English/Spanish-only language restriction; no language restriction is now applied. Records in languages the team does not command are professionally or AI-translated at full-text screening, with translations archived in OSF for auditability. | To avoid excluding potentially eligible non-English/non-Spanish studies and to mitigate language-related publication bias (protocol §15a). |
| v1.7 | Eligibility — Study designs (protocol §8a) | Broadened eligible study designs from qualitative/mixed-methods-only (v1.0-v1.6) to also include quantitative designs (randomized controlled trials, quasi-experimental, single-case, and descriptive/correlational designs). | Full-text screening (Phase 2) revealed that the qualitative/mixed-methods-only criterion was systematically excluding methodologically rigorous, field-foundational studies (e.g., Doyle et al., 2023). |
| v1.7 | Data synthesis — Synthesis strategy (protocol §14a) | Adopted a convergent segregated mixed-methods synthesis design (Stern et al., 2020) in place of a purely qualitative thematic synthesis. | Necessitated by the broadened study-design eligibility (same amendment round); qualitative and quantitative evidence are synthesized separately and integrated in a final configurative step. |

Table S7. For each review finding: contributing studies, assessment across the four GRADE-CERQual components (methodological limitations, coherence, adequacy of data, relevance), and overall confidence level (High / Moderate / Low / Very Low).
